# Synthesis of NiMn-LDH Nanosheet@Ni_3_S_2_ Nanorod Hybrid Structures for Supercapacitor Electrode Materials with Ultrahigh Specific Capacitance

**DOI:** 10.1038/s41598-018-23642-6

**Published:** 2018-03-27

**Authors:** Shuai Yu, Yingxi Zhang, Gaobo Lou, Yatao Wu, Xinqiang Zhu, Hao Chen, Zhehong Shen, Shenyuan Fu, Binfu Bao, Limin Wu

**Affiliations:** 1 Faculty of Engineering and Key Laboratory of Wood Science and Technology of Zhejiang Province, Zhejiang A&F University, Hangzhou, 311300 PR China; 2 Department of Materials Science, Fudan University, Shanghai, 200433 PR China; 3 Collaborative Innovation Center of Novel Organic Chemical Materials of Hubei Province, College of Chemistry and Chemical Engineering, Hubei University, Wuhan, 430062 PR China

## Abstract

One of the key challenges for pseudocapacitive electrode materials with highly effective capacitance output and future practical applications is how to rationally construct hierarchical and ordered hybrid nanoarchitecture through the simple process. Herein, we design and synthesize a novel NiMn-layered double hydroxide nanosheet@Ni_3_S_2_ nanorod hybrid array supported on porous nickel foam *via* a one-pot hydrothermal method. Benefited from the ultrathin and rough nature, the well-defined porous structure of the hybrid array, as well as the synergetic effect between NiMn-layered double hydroxide nanosheets and Ni_3_S_2_ nanorods, the as-fabricated hybrid array-based electrode exhibits an ultrahigh specific capacitance of 2703 F g^−1^ at 3 A g^−1^. Moreover, the asymmetric supercapacitor with this hybrid array as a positive electrode and wood-derived activated carbon as a negative electrode demonstrates high energy density (57 Wh Kg^−1^ at 738 W Kg^−1^) and very good electrochemical cycling stability.

## Introduction

Development of highly efficient energy storage devices is urgently needed to meet the increasing requirement for electrical energy supply in daily life. As one of the most promising types of energy storage devices, supercapacitors (SCs) have been applied in the fields of back-up power systems, portable electronics, telecommunications, vehicles, and so on, for storing the intermittent electrical energy^1–3^. As the key contributor of energy storage of SCs, electrode materials strongly affect the development of SCs, thereby are attracting considerable research interests^3–8^. Pseudocapacitive metal oxides/hydroxides (*e.g*., MnO_2_, NiO, Ni(OH)_2_, Co_3_O_4_, Co(OH)_2_, NiCo_2_O_4_) have been extensively built for use in supercapacitors over the past decades, due to their high energy densities and specific capacitances obtained through fast and reversible faradaic reactions^9–16^. Recently, transitional metallic layered double hydroxides (LDHs), such as NiAl-LDH, NiCo-LDH, and NiMn-LDH, have attracted much attention in supercapacitor electrode materials, owing to their low cost, tunable compositions, layered structures, and potential high electrochemical activities^17–21^. In most cases, however, their unoptimized micro-nanostructure and poor conductivity may lead to insufficient electrolyte access and sluggish charge separation, which in turn hinder the capacitance output of these LDHs^22,23^. Two elegant strategies have been proposed recently to work out the above problems: One is to build up an integrated hierarchical architecture *via* self-assembling LDHs onto specific carbon backbones such as carbon nanotubes, graphene, in which carbon skeletons can not only contribute certain electric double-layer capacitance but also improve the dispersion and electron transport of LDHs, and hence to realize the sufficient utilization of LDHs active materials^17,18,20^. For instance, the NiAl-LDH/reduced graphene oxide superlattice prepared by self-assembly at an atomic scale exhibited two times higher capacity versus pristine NiAl-LDH when used as the active materials for alkaline hybrid supercapacitor^20^. Another strategy is to incorporate highly conductive core materials for LDHs to directly synthesize active materials on the surfaces of current-collectors, and thereby to improve the electron transport among active materials and the electric contacts between active materials and current-collectors^24,25^. For example, Sekhar *et al*. fabricated an Ag nanowires/NiCo-LDH core/shell material supported on carbon cloth by an electrochemical deposition method. Its electrode displayed 2.3 times higher areal capacitance than that without Ag nanowires^24^.

Motivated by these interesting works above, herein, we design and fabricate a novel nickel foam-supported hybrid array consisting of NiMn-LDH nanosheets into which Ni_3_S_2_ nanorods are merged as the skeleton (denoted as NiMn-LDH@Ni_3_S_2_). In this unique structure, relatively high electronic conductivity of Ni_3_S_2_ nanorods can promote the electron transport of NiMn-LDH nanosheets^26–28^, and sulfur ions can block the disintegration of the structure by the elongation between layers^29^, while highly hydroxylated surfaces of LDH nanosheets can improve the wetting ability of Ni_3_S_2_ nanorods to electrolyte. Meanwhile, the rough surfaces of ultrathin nanosheet@nanorod hybrid structures and the well-defined porous structure of the hybrid array favor better exposure of active sites and easier electrolyte access. Moreover, the direct growth on nickel foam current-collector can avoid the electrical resistance of the binder and promote fast electron transport to current collector. Accordingly, the electrode based on the as-obtained NiMn-LDH@Ni_3_S_2_ hybrid array exhibits ultrahigh specific capacitance (2703 F g^−1^) and energy density (68 Wh Kg^−1^), which are significantly higher than those of most reported NiMn-LDH- or Ni_3_S_2_- based active materials as electrodes. And its asymmetric supercapacitor reveals an energy density up to 57 Wh Kg^−1^ at 738 W Kg^−1^ with a long cycling life, which also outperforms those of most the previously asymmetric supercapacitors based on NiMn-LDH or Ni_3_S_2_ derived active materials. This suggests that the NiMn-LDH nanosheet@Ni_3_S_2_ nanorod hybrid array we present here is a very promising for fabrication of high-performance supercapacitors.

## Results and Discussion

### Composition and morphology of hybrid structure

**Figure 1:**
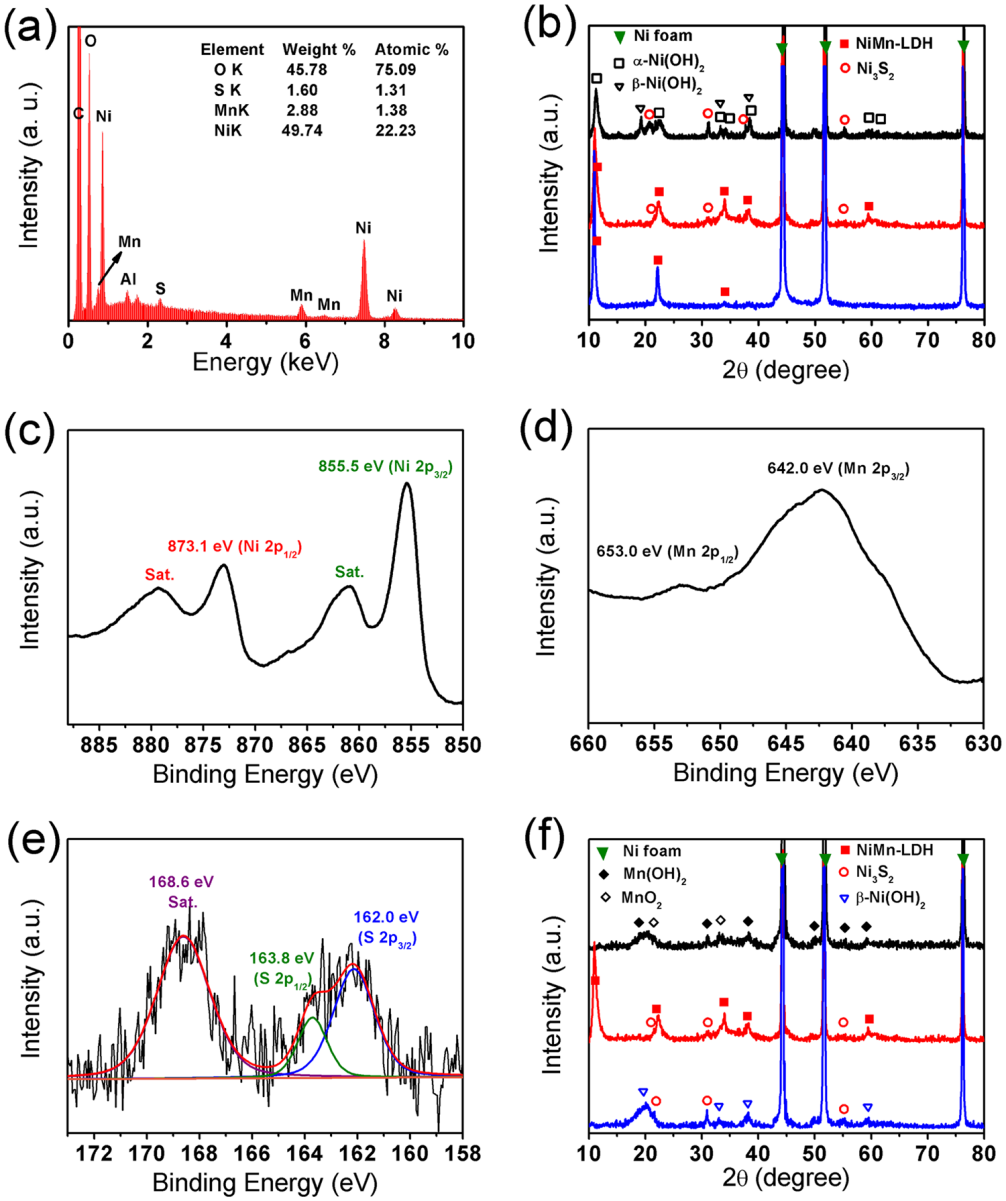
(**a**) EDX spectrum of the hybrid structure powder. (**b**) Comparison of XRD patterns of samples supported on Ni foam obtained at 0.7:0.35 Ni:Mn with thiourea (upper line), urea plus thiourea (middle line) and urea (lower line). (**c**) Ni 2p XPS, (**d**) Mn 2p XPS and (**e**) S 2p XPS of the hybrid structures supported on Ni foam, which was prepared with urea plus thiourea at 0.7:0.35 of Ni:Mn feeding mole ratio. (**f**) Comparison of XRD patterns of samples supported on Ni foam prepared with urea and thiourea at the Ni:Mn feeding mole ratios of 0:1.05 (upper line), 0.7:0.35 (middle line) and 1.05:0 (lower line). (Heating process: 90 ^o^C 4 h + 110 ^o^C 10 h).

The Energy-dispersive X-ray spectroscopy (EDX) spectrum in Fig. 1a shows the presence of O, S, Mn and Ni elements in the as-prepared hybrid materials, except for the C and Al elements from the conductive adhesive carbon tape coated on scanning electron microscope (SEM) aluminum sample holder. The middle line in Fig. 1b demonstrates the typical X-ray diffraction (XRD) pattern of the sample obtained with urea plus thiourea as anion sources. Except for the three sharp peaks originating from the Ni foam substrate, the sample has five well-defined diffraction peaks located at 2*θ* values of 10.9, 22.1, 34.0, 38.0, and 59.5°, which can be successfully indexed to (003), (006), (012), (015) and (110) plane reflections of hydrotalcite-like LDH phase with an average interplanar spacing (d_003_) of 8.11 Å^30,31^, indicating NiMn-LDH structure. In addition, this sample also displays three weaker peaks at 21.5, 31.0, and 55.2°, corresponding to the (101), (110) and (122) plane reflections of Ni_3_S_2_ (JCPDS 44-1418). Thus, the sample obtained with urea plus thiourea as anion sources should be composed of NiMn-LDH and Ni_3_S_2_. The full X-ray photoelectron spectroscopy (XPS) spectrum further proves the basic element composition of as-obtained hybrid material, Ni, Mn, O, and S elements (Supplementary Figure S1a middle line). In Ni 2p XPS spectrum of hybrid material, two shakeup satellites (indicated as “Sat.”) close to two spin-orbit doublets at 873.1 and 855.5 eV are here given as Ni 2p_1/2_ and Ni 2p_3/2_ signals (Fig. 1c)_,_ respectively, suggesting the existence of Ni^2+^ state^32–34^. The Mn 2p XPS spectrum displays Mn 2p_3/2_ and Mn 2p_1/2_ spin-orbit peaks at 642.0 and 653.0 eV (Fig. 1d), indicating the appearance of Mn^3+^ in as-obtained sample^32^. In the S 2p XPS spectrum (Fig. 1e), the peaks at 162.0 and 163.8 eV with the addition of a satellite peak at 168.6 eV can be assigned to S signal in Ni_3_S_2_ phase^35^. Moreover, the Raman spectrum of as-prepared hybrid material (Figure S1b) displays three peaks at 345, 543 and 1040 cm^−1^, which can be assigned to the characteristic bands of Ni_3_S_2_, the metal–oxygen–metal (M–O–M) bonds of NiMn-LDH, and the CO_3_^2–^ in the interlayers of NiMn-LDH, repectively^36,37^. All these results indicate that the NiMn-LDH@Ni_3_S_2_ hybrid structure has been indeed formed. In addition, Fig. 1b and Figure S1c also compare the XRD patterns of the samples supported on Ni foam obtained with thiourea (upper line), urea plus thiourea (middle line) and urea (lower line) as anion sources, respectively. Based on these XRD patterns, the sample prepared with pure thiourea as the anion source is identified as a composite material composed of α-Ni(OH)_2_ (JCPDS 38-0715), β-Ni(OH)_2_ (JCPDS 14-0117), and Ni_3_S_2_ (JCPDS 44-1418). The absence of Mn may be attributed to the Mn element embed into the crystal lattice of nickel-based phases. Meanwhile, the sample synthesized with individual urea as the anion source is found to be pure NiMn-LDH. These results suggest that both urea and thiourea play important roles in the synthesis of NiMn-LDH@Ni_3_S_2_ hybrid arrays. Furthermore, Fig. 1f displays the XRD patterns of the samples supported on Ni foam prepared with individual manganese ions (upper line), nickel-manganese ions (middle line) and pure nickel ions (lower line), respectively. Without Ni^2+^ addition, the as-synthesized product can be identified as a mixture of Mn(OH)_2_ (JCPDS 18-0787) and MnO_2_ (JCPDS 42-1316) phases by XRD pattern, while the sample prepared without Mn^2+^ was found to be composed of β-Ni(OH)_2_ (JCPDS 14-0117) and Ni_3_S_2_ (JCPDS 44-1418) phases.

**Figure 2:**
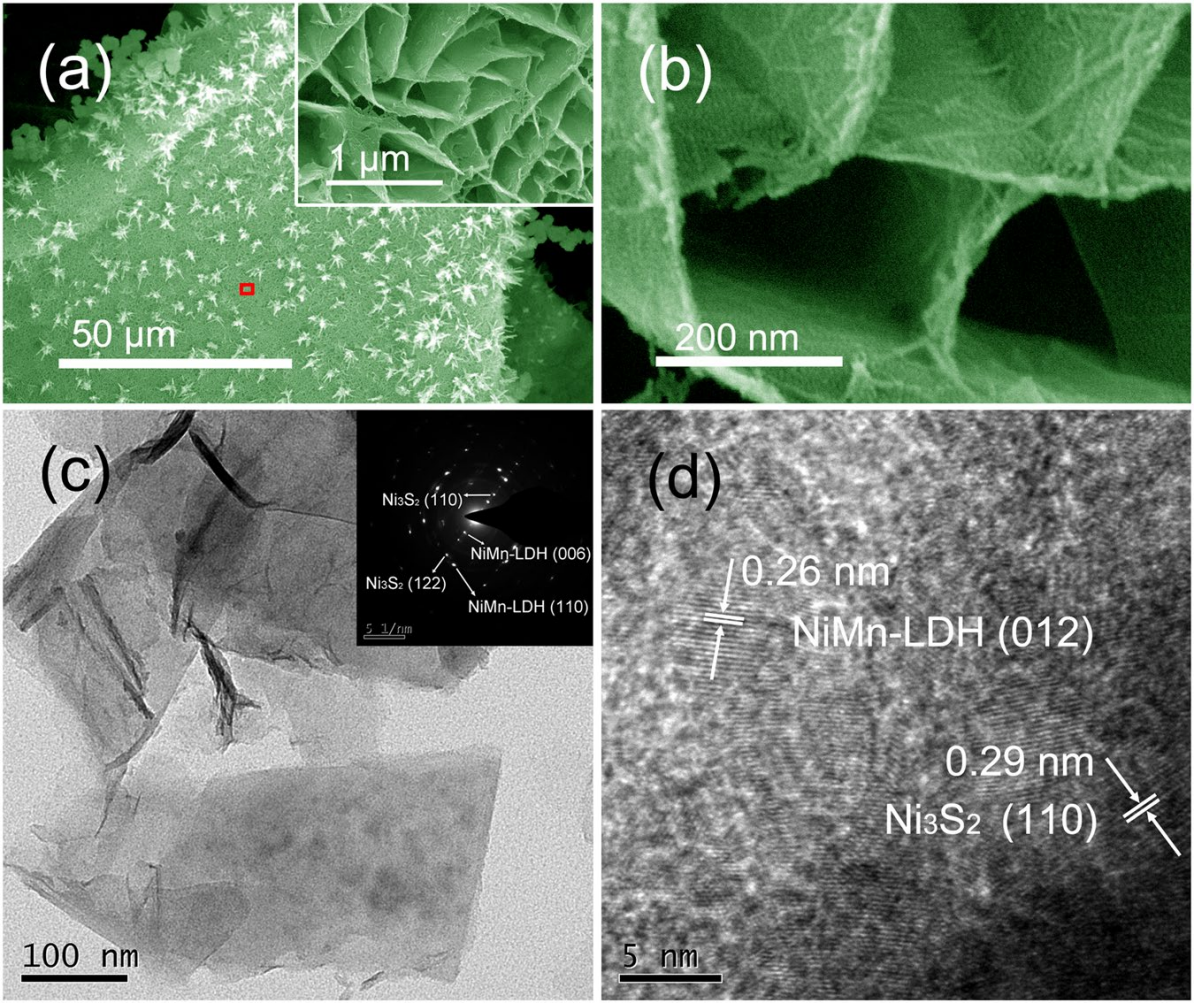
Typical SEM images of (**a**) the hybrid arrays supported on Ni foam, the inset and (**b**) are the high magnification images of the red box area in (**a**). (**c**) Typical TEM image of the hybrid structures, the inset is the SAED pattern of the whole area. (**d**) A HRTEM image of the hybrid structures. (Urea plus thiourea, Ni:Mn: 0.7:0.35, 90 ^o^C for 4 h + 110 ^o^C for 10 h).

Figure 2a shows that the nickel foam skeletons are uniformly covered with hierarchically structured materials, which can be further confirmed by SEM elemental mappings (Supplementary Figure S2a). This product contains two parts. (i) The white aggregates on the surface seem to be composed of short nanorods with the diameter of 6–20 nm (Figure S2b). Based on the EDX spectrum and elemental mappings of a typical aggregate (Figures S2c,d), it can be deduced that its main ingredient should be Ni_3_S_2_. (ii) The main active materials covering on the nickel skeleton are identified as vertically aligned nanosheets with the thickness of around 10 nm as indicated by the inset of Fig. 2a. Notably, these nanosheets are in the form of interlocked arrays with obvious space among adjacent nanosheets. Interestingly, the SEM image at higher-magnification reveals that there also exist some short nanorods attached to the surfaces of nanosheets (Fig. 2b). And most nanorods have been merged into nanosheets as the skeleton of nanosheets, producing rough surfaces.

Typical TEM images further manifest the as-obtained hybrid arrays contain nearly transparent ultrathin nanosheets and well-dispersed nanorods (Fig. 2c and Supplementary Figure S3a). The reflection spots from (110) and (122) crystal planes of Ni_3_S_2_ and (006) and (110) crystal planes of NiMn-LDH can be clearly observed in the inset of Fig. 2c. Meanwhile, two sets of orderly arranged spots with hexagonal-like symmetry is found in the SAED pattern of typical nanorod (the inset of Supplementary Figure S3b), which can be ascribed to the reflections from (110) and (122) crystal planes of Ni_3_S_2_. The lattice fringes with average interplanar spacing of 0.26 and 0.29 nm in the high-resolution TEM (HRTEM) image of hybrid material correspond well to the (012) plane of NiMn-LDH and the (110) plane of Ni_3_S_2_, respectively (Fig. 2d). These results further prove that the nanosheets in hybrid structures should be NiMn-LDH, while the nanorods that coexist with nanosheets are Ni_3_S_2_. In addition, the powder of this hybrid structure is found to have a BET surface area of 56.6 m^2^ g^−1^ and an average pore diameter of 15.8 nm, respectively (Supplementary Figure S4). This novel NiMn-LDH nanosheet@Ni_3_S_2_ nanorod hybrid arrays have not been reported based on the best of our knowledge, and are expected to have excellent electrochemical activity.

### Formation mechanism of hybrid structures

**Figure 3:**
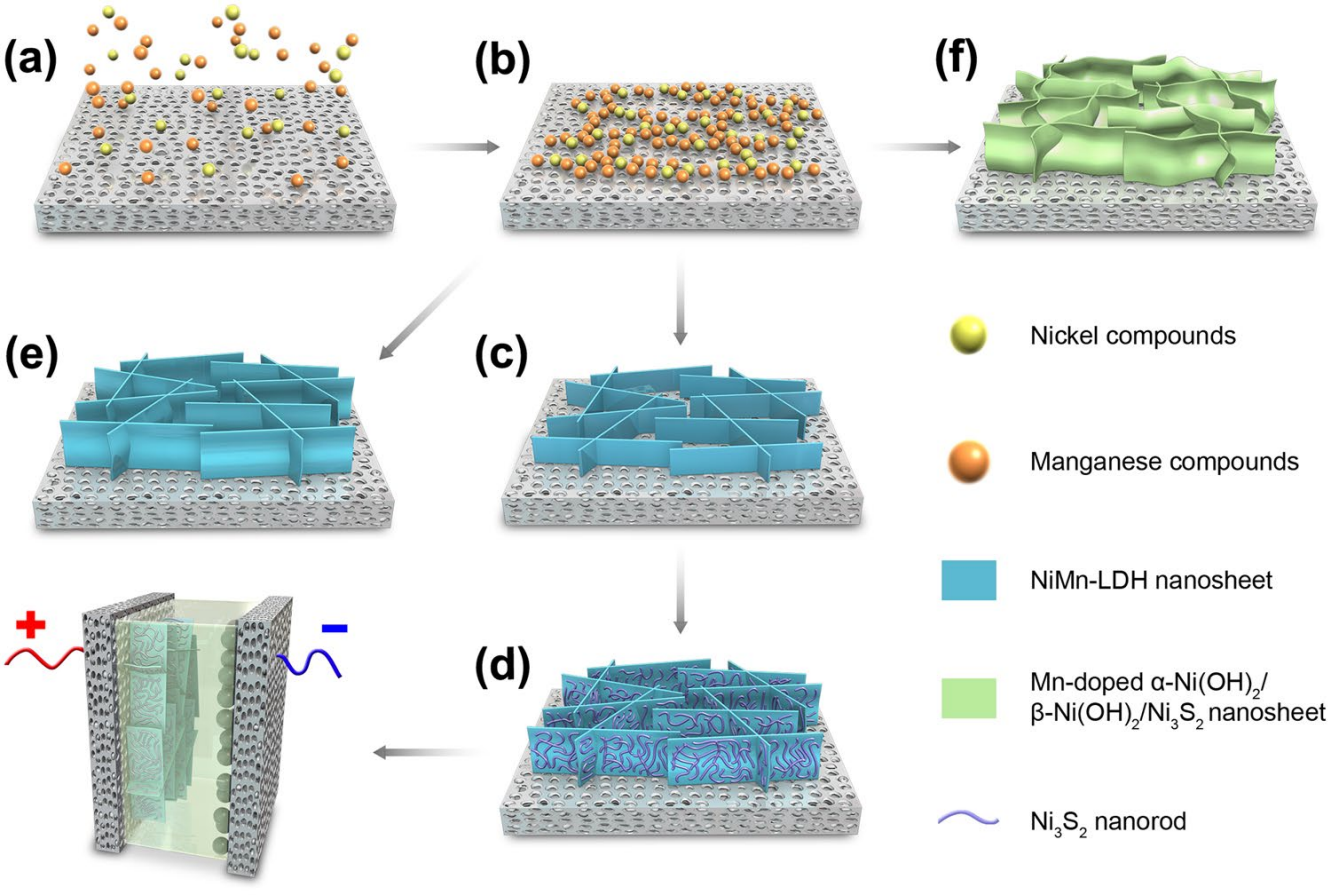
Formation mechanisms of the hybrid structures on Ni foam. (A relatively simple structure is used in this schematic diagram to increase readability. It doesn’t mean that the arrays only grow on the top surface).

Since the NiMn-LDH phase is dominant indicated by the XRD pattern (middle line of Fig. 1b), the hybrid structures probably form based on a mechanism similar to the preferentially oriented growth of LDH nanosheets described in our and others’ previous reports^19,38^. As illustrated by the step a-b-c-d in Fig. 3, at 90 ^o^C processing temperature, Ni^2+^ and Mn^2+^ first react with OH^−^ ions to produce nickel and manganese hydroxides, which precipitate and grow into the primary particles. These primary particles rapidly aggregate into chains which partly deposit on the surface of the nickel substrate to become the aggregation cores. As the other primary particles continue to aggregate, an olation reaction occurs between these particles due to the random dispersion of nickel and manganese hydroxide primary particles. And then they begin to crystallize and grow along the c-axis, gradually forming NiMn-LDH nanosheets^39,40^. In the SEM image of the intermediate product obtained after 90 °C for 0.5 h (Supplementary Figure S5a), very small nanosheets are found to have formed. Moreover, the high-magnification image displays that these small sheets should origin from the chain-like aggregates on the nickel substrate (Figure S5b). After 1 h, the as-obtained intermediate product is identified as some interlaced nanosheets with medium size and poor orientation (Figure S5c). The orientation of nanosheets is improved after 4 h of reaction, and the sheet size increases visibly (Figure S5d). These results well support the proposed formation mechanism above. Until this stage, only NiMn-LDH nanosheets form, that is confirmed by the XRD result of this intermediate product (Supplementary Figure S6a). Because the solubility constant (*K*_*sp*_) of Mn(OH)_2_ is three orders higher than that of Ni(OH)_2_^41^, thus the ratio of Ni to Mn (16.1:1) in the formed NiMn-LDH is far higher than the initial feeding ratio (2.0:1). In addition, owing to lower solubility of Ni_3_S_2_, the S^2−^ ions released by the hydrolysis of thiourea further react with outer surfaces of NiMn-LDH nanosheets to produce Ni_3_S_2_ particles at 110 ^o^C reaction temperature. Because there isn’t obvious difference in the solubility between manganese hydroxide and sulfide, very few Mn^2+^ ions in the formed LDH can combine with S^2−^ ions to synthesize manganese sulfide^41^. As the progress of reaction, these Ni_3_S_2_ particles begin to aggregate, crystallize, and form Ni_3_S_2_ nanorods attached to Ni-Mn LDH nanosheets. This mechanism is also well confirmed by the SEM observation results. In the SEM image of the intermediate product prepared by a reaction of 90 ^o^C for 4 h plus 110 ^o^C for 3 h, only a small amount of nanorods are visible on the surface of nanosheets, yet the amount and size of nanorods obviously increase in the final product (Figures S5e,f). If no thiourea was added (Fig. 3, step a-b-e), the final product is pure NiMn-LDH nanosheets (Supplementary Figure S7a), without Ni_3_S_2_ nanorods due to the lack of S^2−^ ions. When only thiourea was employed as anion source (Fig. 3, step a-b-f), both OH^−^ and S^2−^ ions can be produced. However, because the concentration of OH^−^ ions is relatively low, some of the formed hydroxides turn into sulfides immediately without adequately oriented growth, thereby leading to a high ratio of Ni_3_S_2_ and a poor orientation of formed nanosheets, which are found to be curled, and partial pore structures are blocked (Figure S7b).

### The effect of Ni:Mn feeding mole ratios

**Figure 4:**
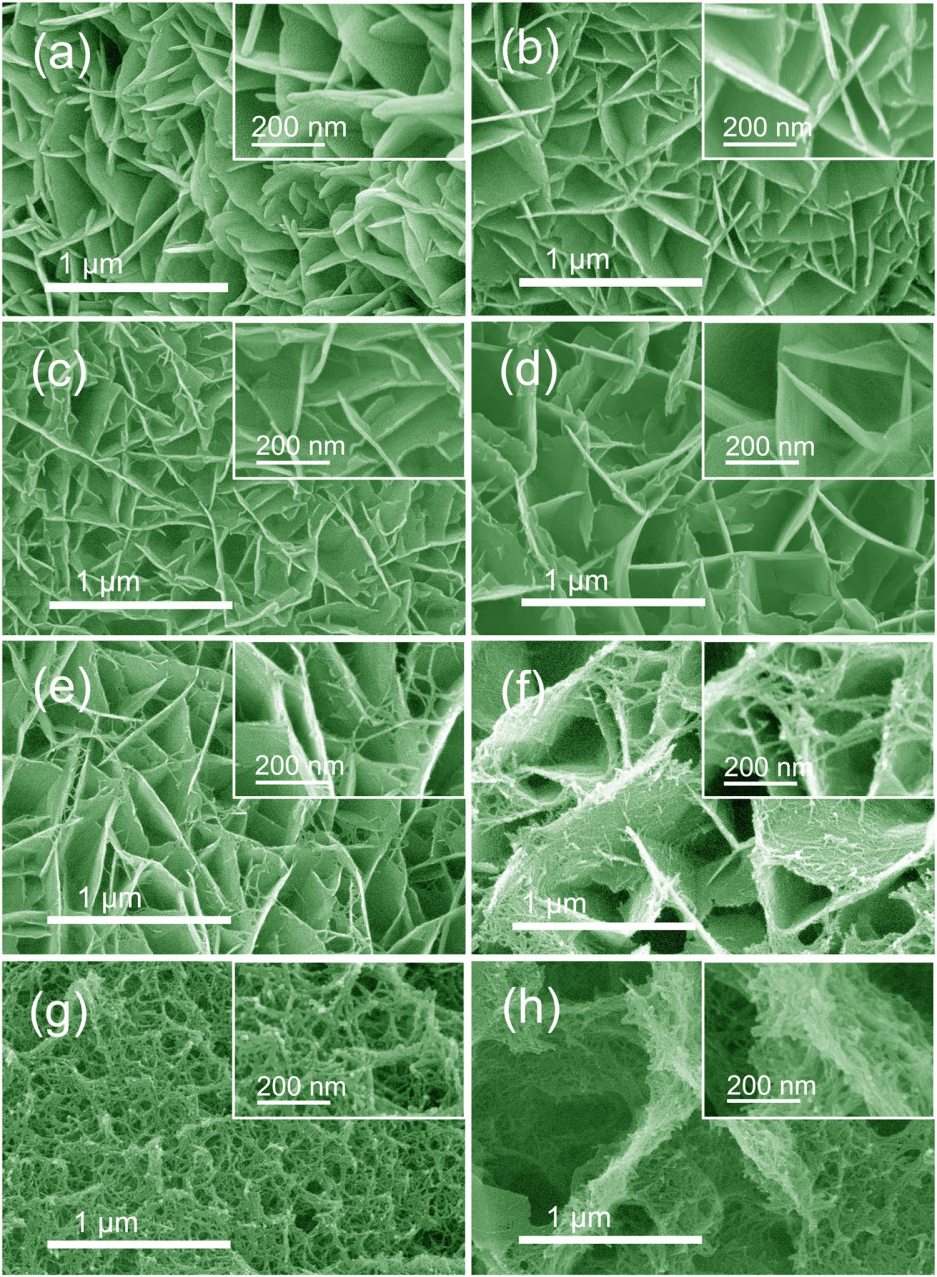
SEM images of the hybrid structures supported on Ni foams prepared with urea plus thiourea at different Ni:Mn feeding mole ratios: (**a**) 0:1.05, (**b**) 0.3:0.75, (**c**) 0.4:0.65, (**d**) 0.5:0.55, (**e**) 0.6:0.45, (**f**) 0.8:0.25, (**g**) 0.9:0.15, (**h**) 1.05:0. The insets are the images of samples at higher magnification. (Heating process: 90 ^o^C for 4 h + 110 ^o^C for 10 h).

Figure 4 further shows the morphology of the as-obtained hybrid structures as a function of the feeding mole ratios of Ni:Mn. Without Ni^2+^, the active material grown on the Ni foam is found to be Mn(OH)_2_/MnO_2_ nanosheets with a mean sheet thickness of 18 nm (Fig. 4a), and some large square particles with the size above 1 μm on the surfaces of nanosheet array (Supplementary Figure S8a). With the addition of Ni^2+^, the mean thickness of the hybrid nanosheets decreases to 15 nm, at a Ni:Mn ratio of 0.3:0.75 (Fig. 4b), and some nanorods appeared in addition to aggregated square particles (Figure S8b). As the ratio of Ni:Mn increases from 0.4:0.65, 0.5:0.55, 0.6:0.45, to 0.7:0.35, more Ni^2+^ are embedded into hybrid nanosheets, and the mean thickness of the hybrid nanosheets decreases to 14, 12, 11, and 10 nm, respectively (Figs 2b and 4c,e). More importantly, incorporation of Ni can induce the growth of Ni_3_S_2_ nanorods to cause the formation of nanosheet@nanorod hybrid arrays. However, with the further increase of Ni:Mn ratio, nanosheet structures trend to degrade, and nanorod products begin to be dominant (Fig. 4f,g). In addition, the white structures on the hybrid array surfaces gradually evolve into nanorods, as the Ni:Mn ratio increases (Figures S8c,g). Once no Mn^2+^ added, only the aggregation of β-Ni(OH)_2_/Ni_3_S_2_ nanorods can be observed (Fig. 4h). Therefore, only the coexistence of Ni^2+^ and Mn^2+^ can induce active material to form LDH phase with the well-defined nanosheet structure. Moreover, under the current preparation process, the incorporation of Mn^2+^ can depress the excessive sulfidation of NiMn-LDH nanosheets by preventing contact between Ni^2+^ and S^2−^ to some degree, thereby avoiding the formation of pure nanorods. Thus, the nanosheet@nanorod hybrid structure can be maintained.

### Electrochemical properties of hybrid electrodes

**Figure 5:**
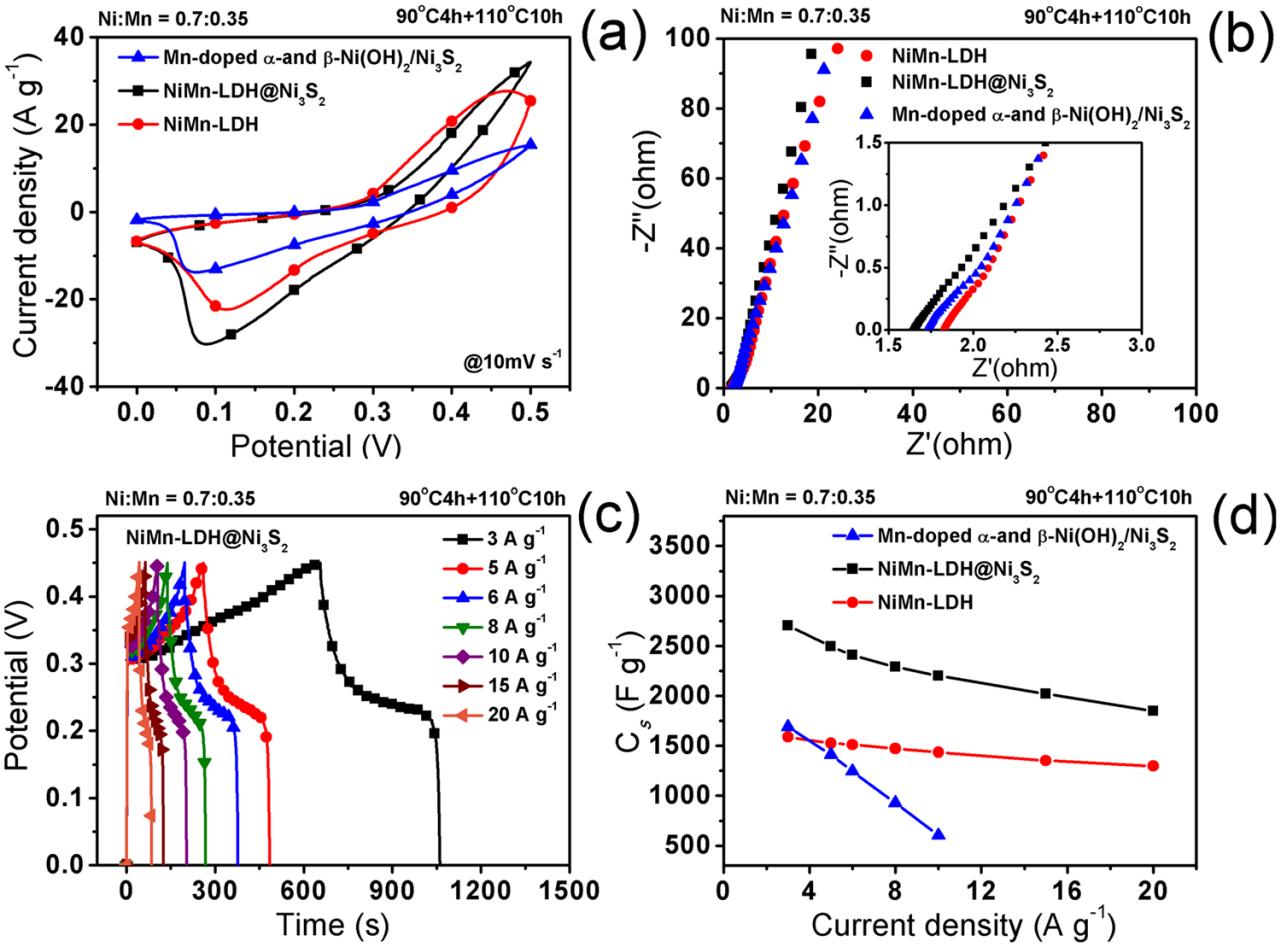
Comparisons of (**a**) CV curves, (**b**) Nyquist plots and (**d**) specific capacitances of NiMn-LDH@Ni_3_S_2_ hybrid array-, and pure NiMn-LDH- and Mn-doped α-Ni(OH)_2_/β-Ni(OH)_2_/Ni_3_S_2_- based electrodes. (**c**) Galvanostatic charge-discharge curves of the hybrid array-electrode at different current densities.

Figure 5a demonstrates the typical cyclic voltammetry (CV) curve of the as-obtained NiMn-LDH nanosheet@Ni_3_S_2_ nanorod hybrid array, which was fabricated under the condition with a 0.7:0.35 feeding mole ratio of Ni:Mn, a heating process of 90 ^o^C for 4 h plus 110 ^o^C for 10 h, supported on Ni foam as an electrode for supercapacitor at a scan rate of 10 mV s^−1^. The CV curves of pure NiMn-LDH and Mn-doped α-Ni(OH)_2_/β-Ni(OH)_2_/Ni_3_S_2_ electrodes were also measured for the sake of comparison. Well-defined redox peaks within 0-0.5 V are associated with the faradaic redox reactions related to M-O/M-O-OC and M-S/M-S-OC (M represents Ni or Mn, C represents H or K)^42–44^, indicating the strong pseudocapacitive nature of these electrodes. Because the specific capacitance (*C*_*s*_) is proportional to the average area of a CV curve, the comparison of CV curves, as shown in Fig. 5a, further indicates that the hybrid array-based electrode possesses a significantly higher *C*_*s*_ than pure NiMn-LDH- and Mn-doped α-Ni(OH)_2_/β-Ni(OH)_2_/Ni_3_S_2_- based electrodes^45^. This can be attributed to the unique morphology of the NiMn-LDH nanosheet@Ni_3_S_2_ nanorod hybrid electrode, especially the interconnected porous structure, ultrathin nanosheets, and pretty rough surfaces can provide more active sites for efficient exposure to electrolyte for better electrochemical reactions. Furthermore, the NiMn-LDH@Ni_3_S_2_ hybrid electrode also exhibits lower equivalent series resistance (*R*_*s*_, the real axis intercept in Fig. 5b), implying better electron transportation from active material to the current collector.

The galvanostatic charge-discharge curves of the NiMn-LDH@Ni_3_S_2_ hybrid array-based electrode at different current densities show strong pseudocapacitive behavior (Fig. 5c). The comparison in *C*_*s*_ based on charge-discharge curves illustrates that the as-prepared NiMn-LDH@Ni_3_S_2_ electrode has nearly increased by 60% in specific capacitances compared to pure NiMn-LDH and Mn-doped α-Ni(OH)_2_/β-Ni(OH)_2_/Ni_3_S_2_ electrodes (Fig. 5d). These results indicate that the incorporation of Ni_3_S_2_ into NiMn-LDH can improve the micro-nanostructure and electron transportation, and thereby significantly enhance the capacitance output.

**Figure 6:**
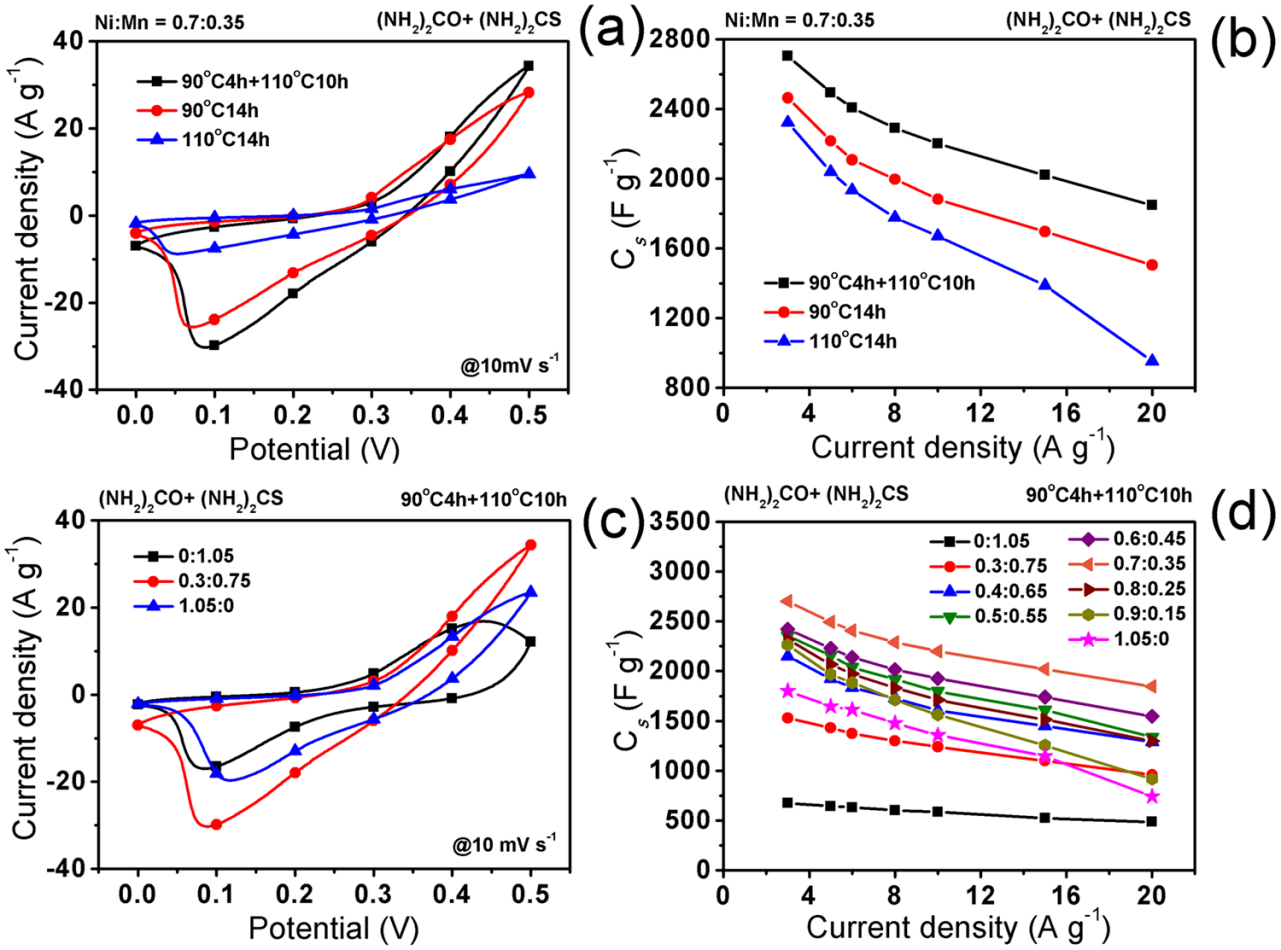
Comparisons of (**a**) CV curves and (**b**) specific capacitances of the hybrid electrodes prepared with urea plus thiourea at different heating processes (Ni:Mn: 0.7:0.35). Comparisons of (**c**) CV curves and (**d**) specific capacitances of the hybrid electrodes prepared with urea plus thiourea at different feeding mole ratios of Ni:Mn (Heating process: 90 ^o^C 4 h + 110 ^o^C 10 h).

The heating process during preparation of the hybrid array also influences the electrochemical properties of the electrodes because it impacts the morphological control of the hybrid structures as follows: When the two-step heating process of 90 ^o^C for 4 h plus 110 ^o^C for 10 h was replaced by a one-step heating process of 90 ^o^C for 14 h during the whole hydrothermal reaction, only aggregated micro-rods consisting of an enormous number of short nanorods were observed (Supplementary Figures S9a,b). When the reaction was executed under a higher temperature of 110 ^o^C for 14 h, the excessive sulfidation of hydroxides also resulted in the formation of pure nanorod aggregates, but these nanorods appeared to be longer (Figures S9c,d). Accordingly, the NiMn-LDH@Ni_3_S_2_ hybrid array-electrode prepared with a heating process of 90 ^o^C for 4 h plus 110 ^o^C for 10 h exhibits much larger CV curve area and higher specific capacitances than the electrodes from the heating processes of 90 ^o^C for 14 h or 110 ^o^C for 14 h, as shown in Fig. 6a,b.

Figure 6c compares the CV curves of Mn(OH)_2_/MnO_2_, β-Ni(OH)_2_/Ni_3_S_2_, and NiMn-LDH@Ni_3_S_2_ electrodes to study the effect of Ni^2+^ and Mn^2+^. It can be seen that the CV area of the NiMn-LDH@Ni_3_S_2_ hybrid array-based electrode is larger than those of the Mn(OH)_2_/MnO_2_ electrode obtained without Ni^2+^ and the β-Ni(OH)_2_/Ni_3_S_2_ electrode, indicating that the coexistence of Ni^2+^ and Mn^2+^ supports the active material to obtain better electrochemical performances. Figure 6d displays the specific capacitances of hybrid electrodes prepared at different Ni:Mn feeding mole ratios. As more Ni^2+^ becomes embedded, *C*_*s*_ increases. However, too high ratio of Ni:Mn, *e.g*., 0.8:0.25, causes a decrease in *C*_*s*_. This *C*_*s*_ variation tendency corresponds to the morphological evolution of hybrid structures as shown in Fig. 4. The nanosheet@nanorod hybrid array formed at a ratio of 0.7:0.35 Ni:Mn exhibits the highest *C*_*s*_ values, with 2703, 2494, 2408, 2290, 2201, 2021, and 1847 F g^−1^ at current densities of 3, 5, 6, 8, 10, 15, and 20 A g^−1^, respectively, in which the highest *C*_*s*_ (2703 F g^−1^ at 3 A g^−1^) is significantly higher than those of most reported NiMn-LDH- and Ni_3_S_2_- based active materials (Supplementary Table S1). Such high specific capacitance is attributed to the unique composition and structure features of the NiMn-LDH@Ni_3_S_2_ hybrid arrays we present here: (i) There is a synergetic effect between NiMn-LDH and Ni_3_S_2_. On the one hand, relatively high electronic conductivity of Ni_3_S_2_ nanorods can enhance the electron transport of NiMn-LDH nanosheets^27,28^. In addition to being illustrated by the EIS result in Fig. 5b, this point is also supported by the following evidence. Compared with those of pure NiMn-LDH (Figures S1d-e lower lines), the Ni 2p and Mn 2p peaks in the XPS spectra (Figures S1d,e middle lines) of NiMn-LDH@Ni_3_S_2_ have obvious position shifts, confirming the existence of strong electron interactions between NiMn-LDH and Ni_3_S_2_^37,46^. On the other hand, highly hydroxylated surfaces of LDH nanosheets can improve the wetting ability of Ni_3_S_2_ nanorods to electrolyte; (ii) The rough surfaces and well-defined porous structure of the nanosheet@nanorod hybrid arrays allow better exposure of active sites and easier electrolyte access; (iii) The direct growth on nickel foam can avoid the electrical resistance of the binder to promote fast electron transport to current collector.

Based on the obtainable *C*_*s*_ values and the reported calculation method^47,48^, the energy densities of the as-obtained best NiMn-LDH@Ni_3_S_2_ hybrid array electrode can be calculated to be 68, 60, 52, 46, 41, 31, and 22 Wh Kg^−1^ at average power densities of 602, 957, 1045, 1284, 1480, 1815, and 1895 W kg^−1^, respectively. In the same way, the maximum energy density (68 Wh kg^−1^ at 602 W kg^−1^) has also exceeded that of some similar electrode materials, such as carbon/NiMn-LDH nanosheets (34.6 Wh kg^−1^ at 89.95 W kg^−1^)^47^, graphene oxide sponge/NiMn layered double oxide nanosheets (46.3 Wh kg^−1^ at 112.6 W kg^−1^)^48^, and Ni_3_S_2_ nanoflakes (44.9 Wh kg^−1^ at 208.3 W kg^−1^)^49^.

### Electrochemical properties of NiMn-LDH@Ni_3_S_2_//AC asymmetric supercapacitors

**Figure 7:**
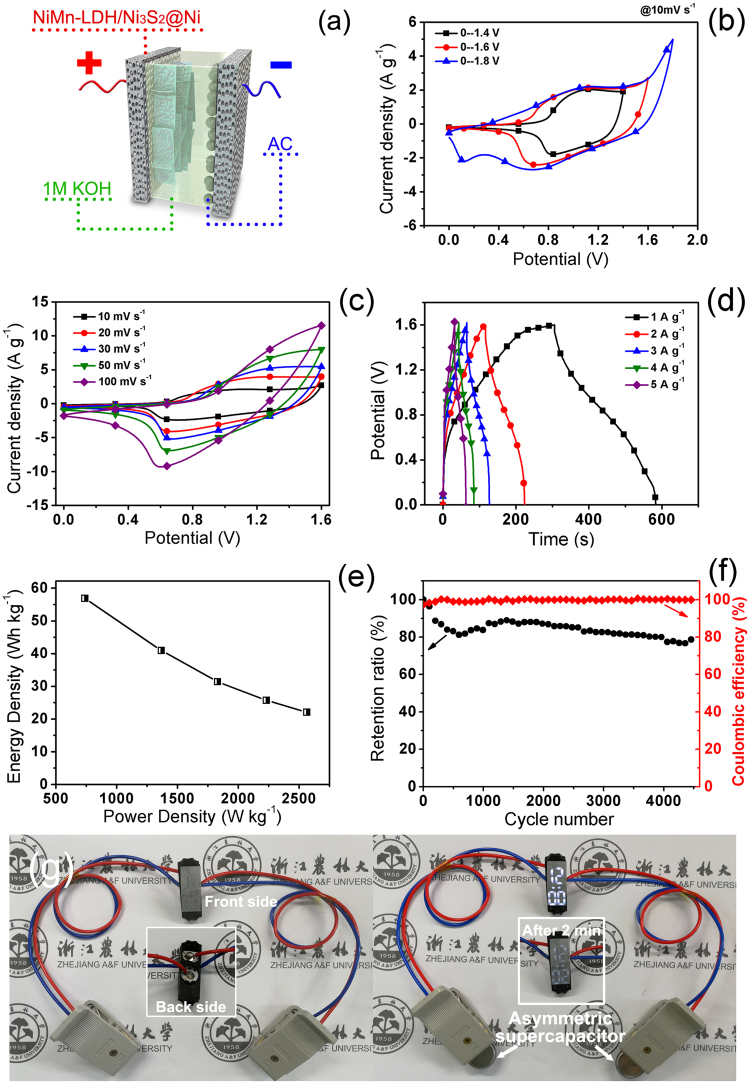
(**a**) Scheme of NiMn-LDH@Ni_3_S_2_//AC asymmetric supercapacitor. CV curves of the asymmetric supercapacitor at (**b**) different electrochemical windows and (**c**) different scan rates. (**d**) Galvanostatic charge-discharge curves, (**e**) energy density vs. power density curve, and (**f**) cycling performances (at 5 A g^−1^) of the asymmetric supercapacitor. (**g**) The photographs of an electronic timer powered by two coin cell asymmetric supercapacitors.

In order to provide a suitable negative material for the NiMn-LDH@Ni_3_S_2_ electrode to construct an asymmetric supercapacitor, we also prepared a wood-derived activated carbon (AC) and investigated its morphology, pore structure, and capacitive performances. This AC owns irregular sharp and visible pore structures (Supplementary Figure S10a), and its BET surface area and average pore diameter can reach up to 701 m^2^ g^−1^ and 3.1 nm, respectively (Figures S10b-c). When served as electrode active materials, the as-obtained AC exhibits excellent electric double-layer capacitance property within −1.0 to 0.0 V (Figure S10d). The *C*_*s*_ of AC-electrode can be calculated from its galvanostatic charge-discharge curves (Figure S10e) and reach up to 364 F g^−1^ at 0.2 A g^−1^ (Figure S10f), which is comparable to those of the previously reported activated carbons (236–400 F g^−1^)^50–54^. Owing to the excellent capacitance properties of the as-obtained NiMn-LDH@Ni_3_S_2_ (within 0.0−0.5 V) and AC (within −1.0–0.0 V), an asymmetric supercapacitor (NiMn-LDH@Ni_3_S_2_//AC) were successfully fabricated with NiMn-LDH@Ni_3_S_2_ and AC as the positive and negative electrode materials, respectively, as illustrated in Fig. 7a. The comparison of CV curves in Fig. 7b indicates the as-fabricated asymmetric supercapacitor exhibits good supercapacitive behavior within different voltage windows. However, the CV profile displays obvious deformation within 0–1.8 V window, implying poor stability of electrode materials in this state. Thus, a moderate electrochemical window of 0−1.6 V was employed to investigate the capacitive performances of as-obtained asymmetric supercapacitor. Figure 7c,d demonstrate that this asymmetric supercapacitor benefits from double contribution of electric double-layer capacitance and pseudocapacitance. Its energy densities can be calculated to be 57, 41, 31, 26, and 22 Wh Kg^−1^ at the average power densities of 738, 1367, 1830, 2231, and 2564 W Kg^−1^, respectively, as shown in Fig. 7e, from its galvanostatic charge-discharge curves (Fig. 7d). The highest energy density of the asymmetric supercapacitor can be found to be 57 Wh Kg^−1^ at the average power density of 738 W Kg^−1^. At a high power density of 2564 W Kg^−1^, the energy density can still remain 22 Wh Kg^−1^. The obtainable maximum energy density has surpassed those of most previously reported NiMn-LDH- and Ni_3_S_2_- based asymmetric supercapacitors (Supplementary Table S2). It is generally known that the electrochemical stability is crucial for commercial energy storage device. Thus, the galvanostatic charge-discharge measurement was also employed to evaluate the cycling stability of the as-fabricated asymmetric supercapacitor. As shown in Fig. 7f, the specific capacitance of the as-obtained asymmetric supercapacitor experiences a rapid decline in the initial 700 cycles, possibly due to the flaking off of Ni_3_S_2_ white aggregates attached to the outer surfaces of the NiMn-LDH@Ni_3_S_2_ electrode (Fig. 2a). Then its *C*_*s*_ increases gradually up to 89% of its initial value, which should be attributed to the full activation of electrode meterials. After that, the *C*_*s*_ decreases again. After 4500 cycles, about 79% of its original capacitance can be retained, which is comparable to some previously reported similar asymmetric supercapacitors (Table S2). The SEM images of the NiMn-LDH@Ni_3_S_2_ electrode after this cycling stability test display that the nanosheet array is preserved (Supplementary Figure S11). However, most surface aggregates have fallen off, and it’s hard to find Ni_3_S_2_ nanorods on the surface of NiMn-LDH nanosheets, possibly due to an irreversible conversion from Ni_3_S_2_ to nickel hydroxides after a long-term electrochemical redox reaction in the alkaline electrolyte^36^. These should be the main reasons for the *C*_*s*_ attenuation. Furthermore, after a few initial cycles, the Coulombic efficiency can maintain about 99% during 4500 cycles, indicating good electrochemical stability of the NiMn-LDH@Ni_3_S_2_//AC asymmetric supercapacitor. After charged for only 30 seconds, two NiMn-LDH@Ni_3_S_2_//AC coin cell asymmetric supercapacitors (19.1 mg active materials) successfully powered a 3 V electronic timer for at least 2 minutes, as displayed in Fig. 7g. These results indicate that the as-prepared NiMn-LDH nanosheet@Ni_3_S_2_ nanorod hybrid array can be considered as a promising candidate for fabrication of practical supercapacitors.

## Conclusions

In summary, a novel NiMn-LDH nanosheet@Ni_3_S_2_ nanorod hybrid array supported on the three-dimensional porous nickel foam has been successfully fabricated using a facile solvothermal co-deposition method involving a two-step heating process and urea plus thiourea as anion sources. Due to the ultrathin and rough nature, the well-defined porous structure of the hybrid array, and the synergetic effect between NiMn-LDH nanosheets and Ni_3_S_2_ nanorods, the electrode based on this novel hybrid array exhibits ultrahigh specific capacitance (2703 F g^−1^), which is significantly higher than those of most reported NiMn-LDH- and Ni_3_S_2_- based active materials. And its asymmetric supercapacitor also displays satisfactory energy density (57 Wh Kg^−1^ at 738 W Kg^−1^), which is considerably superior to those of most previously reported asymmetric supercapacitors based on NiMn-LDH or Ni_3_S_2_, suggesting that the NiMn-LDH nanosheet@Ni_3_S_2_ nanorod hybrid array we present here is a very promising for fabrication of high-performance supercapacitors. This method we present here could be extended to fabricate other LDH/metal sulfide hybrid materials with high electrochemical activity.

## Methods

### Fabrication of Hybrid Materials

In a typical process, the commercial Ni foam was pre-washed successively with acetone, 2 M HCl solution, deionized water, and absolute ethanol, each for 15 min, to ensure a clean surface. The cleaned nickel foam was then immersed in a 100 mL Teflon autoclave with a homogeneous solution of Ni(NO_3_)_2_·6H_2_O (0.7 mmol), Mn(NO_3_)_2_ (0.35 mmol), urea (0.063 g), thiourea (0.08 g), H_2_O (40 mL) and absolute ethanol (30 mL), followed by heating the autoclave in an oven at 90 ^o^C for 4 h and subsequently 110 ^o^C for 10 h. Here, urea and thiourea were used as anion sources to produce OH^−^ and S^2−^ ions by hydrolysis. After cooled down to room temperature, the Ni foam was washed with deionized and absolute ethanol for three times, respectively. Then, it was dried at 60 ^o^C to remove the absorbed solvents. For the sake of comparison, the hybrid materials with pure urea or individual thiourea as anion source, or different heating processes, or different Ni:Mn feeding mole ratios were also fabricated using the similar procedure. The mass of the hybrid material on Ni foam was determined by subtracting the weight before deposition from the weight after deposition. The loading density of active materials was about 2 mg cm^−2^ for all electrodes.

### Synthesis of Wood-Derived Activated Carbon (AC)

Polar wood sawdust (in a corundum crucible) was first pyrolyzed under N_2_ atmosphere at high temperature by applying a tube furnace to prepare wood-derived carbon. The wood sawdust was heated to 200 ^o^C at the heating ramp rate of 3 ^o^C min^−1^ and held at this temperature for 1 h to remove moisture. The sample was then heated to 900 ^o^C at about 3 ^o^C min^−1^ and held at this temperature for 1 h to remove volatile organics and to carbonize solid residues, and then cooled to room temperature, to producing brown-black wood-derived carbon. The as-obtained sample was thoroughly washed with 1 mol L^−1^ HCl to remove soluble inorganic salts and then washed with distilled water and absolute ethanol to neutral followed by drying in a vacuum oven at 100 ^o^C overnight. Subsequently, this powder was thoroughly mixed with KOH solution (10 mol L^−1^) (carbon/KOH, 1:4 by weight ratio) under 100 ^o^C for several hours to produce a black jelly-like slurry, which was then treated by the same pyrolysis process. 2 mol L^−1^ HCl was added to the as-obtained sample to neutralize excess alkali, and then washed with distilled water and absolute ethanol, respectively. By this process the wood-derived activated carbon (AC) was obtained after drying in a vacuum oven at 100 ^o^C overnight.

### Fabrication of AC electrodes

A mixture of AC, 15 wt% of acetylene black (as an electrical conductor), 5 wt% of polytetrafluorene–ethylene (as a binder), and a small amount of water was prepared by milling to produce a homogeneous paste. This paste was then pressed onto nickel foam current-collectors to produce the AC electrode.

### Characterization

The morphologies were observed by scanning electron microscopy (SEM, SU8000, Hitachi). The selected-area electron diffraction (SAED) pattern and transmission electron microscope (TEM) images were obtained on a FEI Tecnai G2 F20 S-TWIN field emission microscope. The crystalline structure was characterized by X-ray diffraction (XRD) patterns recorded in a PANalytical X’pert X-ray diffractometer with Cu Kα radiation. X-ray photoelectron spectroscopy (XPS, Thermo ESCALAB 250XI) and Energy-dispersive X-ray spectroscopy (EDX, TSL, AMETEK) measurements were employed to investigate the elemental compositions of the samples. An automated adsorption apparatus (BELSORP-max) was used to analyze the surface characteristics of the samples.

### Electrochemical measurement

The electrochemical properties of the as-obtained hybrid material- and the AC- based electrodes were investigated under a three-electrode cell configuration at 25 ^o^C in 1 M KOH. The nickel foam supporting hybrid structures acted as the working electrodes, which were soaked in a 1 M KOH solution and degassed in a vacuum for 5 h before the electrochemical test. Platinum foil and a saturated calomel electrode (SCE) were used as the counter and reference electrodes, respectively. The electrochemical properties of asymmetric supercapacitor were investigated under a two-electrode cell configuration with NiMn-LDH@Ni_3_S_2_ and AC as the positive and negative electrode materials, respectively, in 1 M KOH electrolyte solution. The cyclic voltammetry (CV) and electrochemical impedance spectroscopy (EIS) measurements were conducted on a CHI 660E electrochemical workstation (Shanghai CH Instrument Company, China). The galvanostatic charge-discharge tests were carried out on a Land test system (LAND CT2001A, China). The *C*_*s*_, energy and power densities were calculated based on the total mass of active materials.

## Electronic supplementary material

Supporting information
