# Supplementary material for: Synthesis of NiMn-LDH Nanosheet@Ni3S2 Nanorod Hybrid Structures for Supercapacitor Electrode Materials with Ultrahigh Specific Capacitance
Source: Sci Rep. 2018 Mar 27;8:5246. doi: 10.1038/s41598-018-23642-6 (PMC5869735; doi:10.1038/s41598-018-23642-6)
Supplement: Supplementary file 1 — Supporting information [file 41598_2018_23642_MOESM1_ESM.pdf]

Supporting Information

Synthesis of NiMn-LDH Nanosheet@Ni_3S_2 Nanorod Hybrid Structures for Supercapacitor Electrode Materials with Ultrahigh Specific Capacitance

Shuai Yu[a], Yingxi Zhang[a,c], Gaobo Lou[a], Yatao Wu[a], Xinqiang Zhu[a], Hao Chen[a,], Zhehong Shen[a,]*, Shenyuan Fu[a], Binfu Bao[a], and Limin Wu[b,c,]**

*Corresponding author. E-mail: haochen10@fudan.edu.cn (H. Chen); zhehong.shen@vip.163.com (Z. Shen); lmw@fudan.edu.cn (L. Wu).

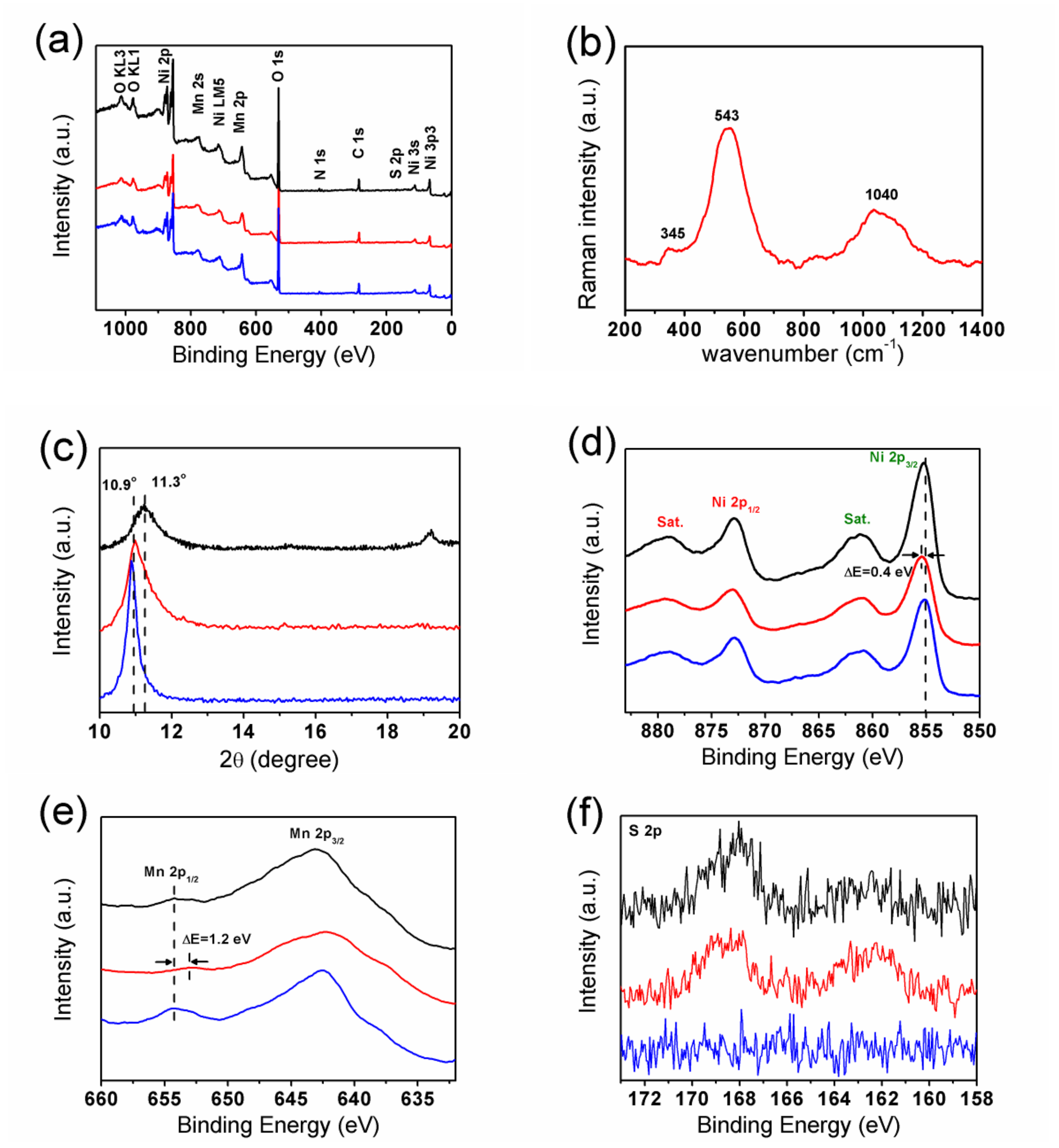

Figure S1. Comparisons of (a) full XPS spectra, (c) XRD patterns, (d) Ni 2p XPS, (e) Mn 2p XPS and (f) S 2p XPS spectra of samples supported on Ni foam obtained at 0.7:0.35 Ni:Mn with thiourea (upper line), urea plus thiourea (middle line) and urea (lower line). (Heating process: 90°C 4 h + 110 ^{o}C 10 h). (b) Raman spectra of the hybrid structures supported on Ni foam (The sample was prepared at 90°C 4 h + 110 ^{o}C 10 h with urea plus thiourea and 0.7:0.35 of Ni:Mn feeding mole ratio.)

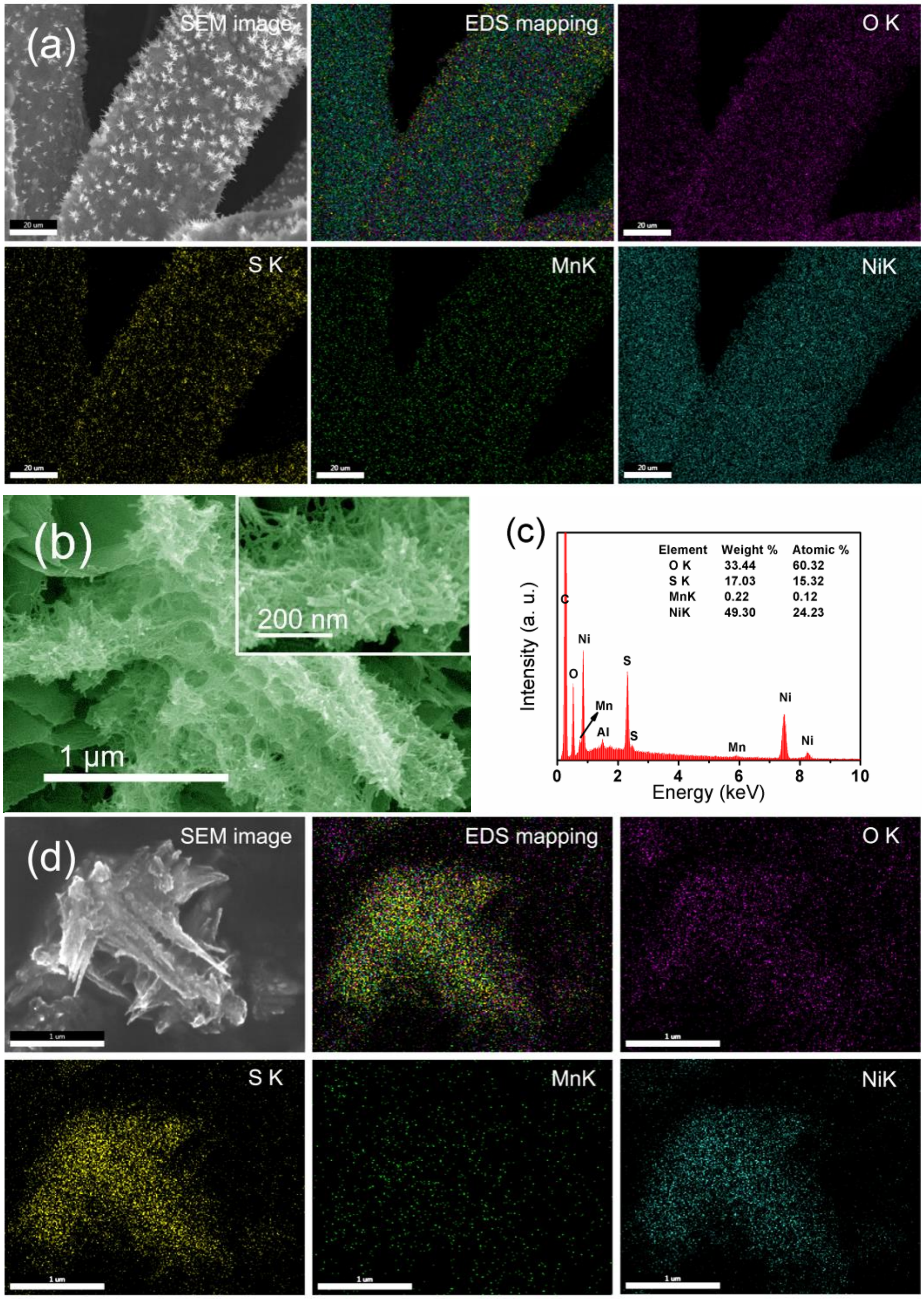

Figure S2. (a) SEM elemental mappings of the sample supported on 3D Ni foam. (b) SEM images at two different magnifications, (c) EDX spectrum, and (d) SEM elemental mappings of a white aggregate. (Urea plus thiourea, Ni:Mn: 0.7:0.35, 90°C 4 h + 110 ^{o}C 10 h).

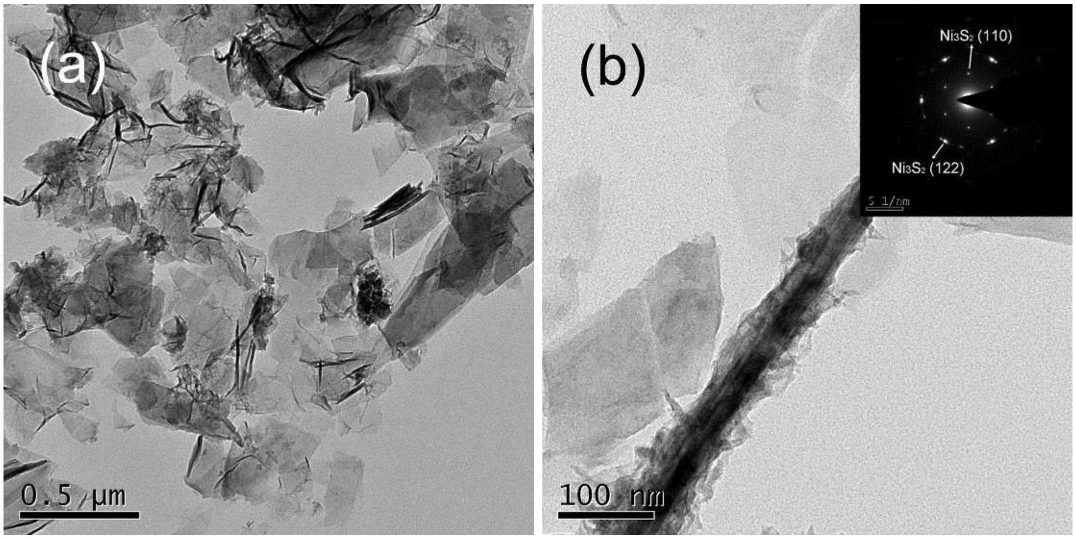

Figure S3. (a) A TEM image of the hybrid structures at a low magnification. (b) A TEM image of the nanorod in the hybrid structures, the inset is the SAED pattern of the whole area. (Urea plus thiourea, Ni:Mn: 0.7:0.35, 90 ^{o}C 4 h + 110 ^{o}C 10 h).

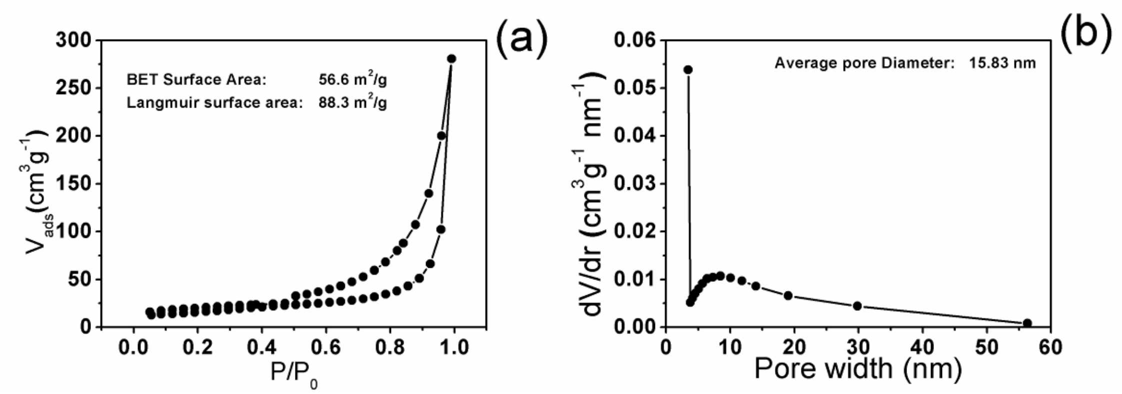

Figure S4. (a) Nitrogen (77 K) adsorption/desorption isotherms and (b) BJH pore size distribution curves of the powder of hybrid structures, which was scraped from Ni foam supported samples. (Urea plus thiourea, Ni:Mn: 0.7:0.35, 90 ^{o}C 4 h + 110 ^{o}C 10 h).

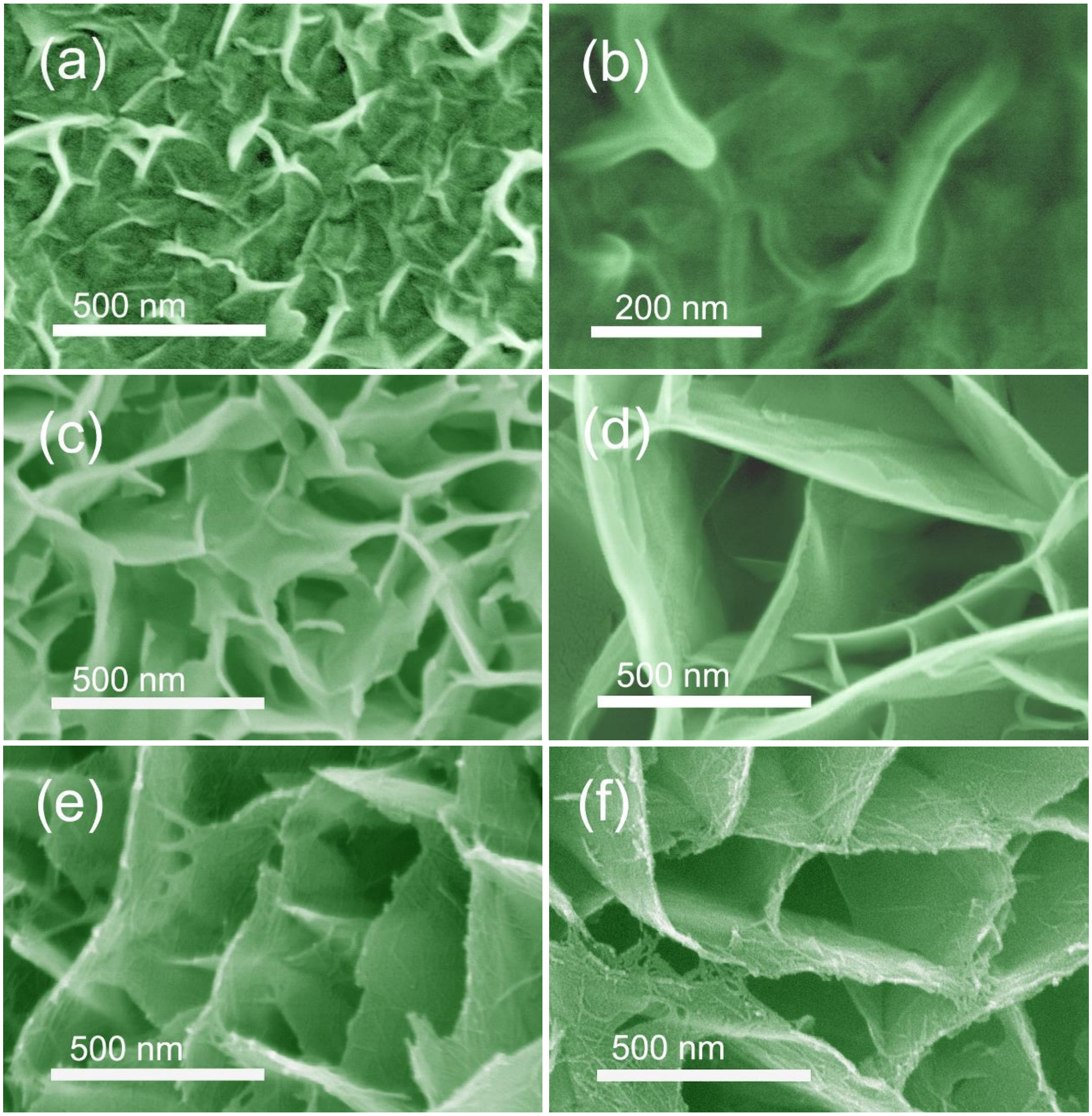

Figure S5. SEM images of the hybrid structures supported on Ni foam prepared with different reaction times: (a, b) 90 ^{o}C 0.5 h at two different magnifications, (c) 90 ^{o}C 1 h, (d) 90 ^{o}C 4 h, (e) 90 ^{o}C 4 h + 110 ^{o}C 3 h, (f) 90 ^{o}C 4 h + 110 ^{o}C 10 h (Urea plus thiourea, Ni:Mn: 0.7:0.35).

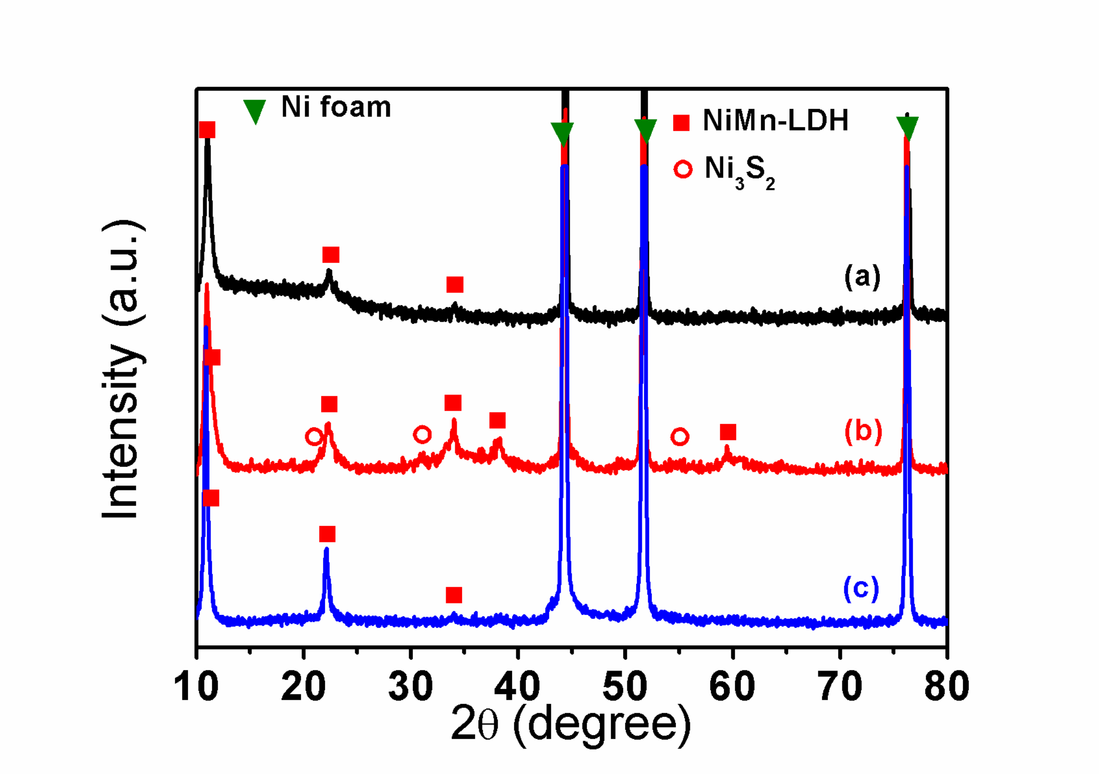

Figure S6. Comparison of XRD patterns of samples supported on Ni foam obtained with different conditions: (a) 0.7:0.35 Ni:Mn, urea plus thiourea, 90°C 4 h, (b) 0.7:0.35 Ni:Mn, urea plus thiourea, 90°C 4 h + 110 °C 10 h, (c) 0.7:0.35 Ni:Mn, individual urea, 90°C 4 h + 110 °C 10 h.

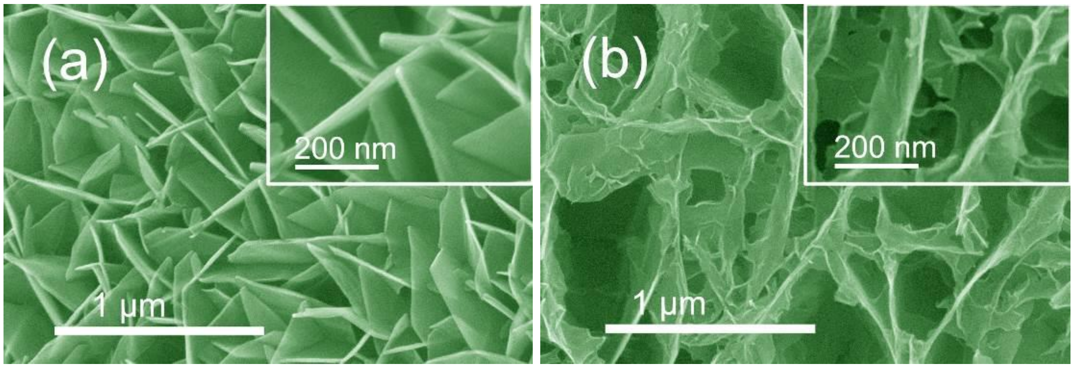

Figure S7. SEM images of the hybrid structures supported on Ni foam prepared with (a) individual urea and (b) individual thiourea (Ni:Mn: 0.7:0.35, 90°C 4 h + 110 °C 10 h). The insets are the images of hybrid structures at higher magnification.

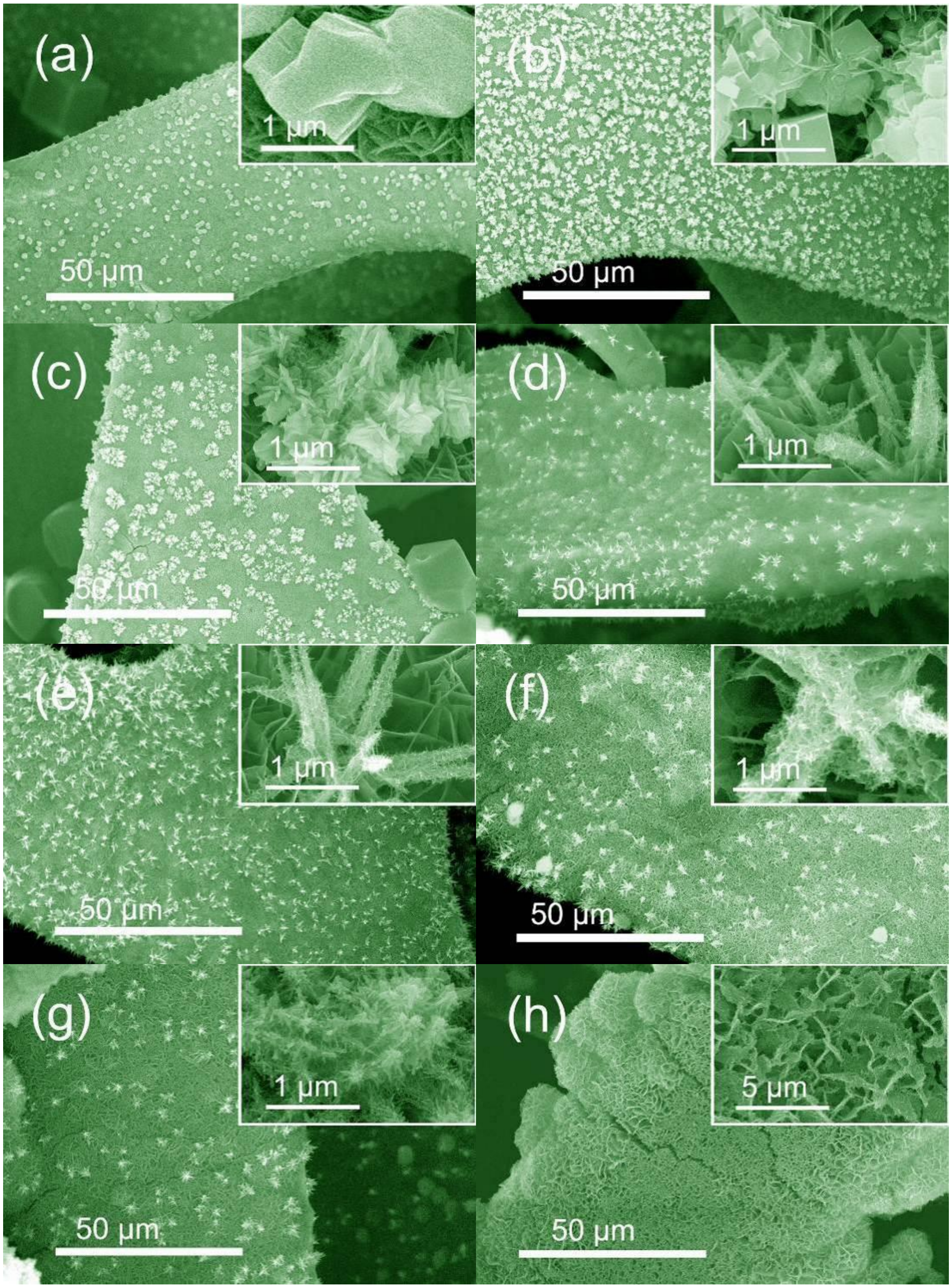

Figure S8. SEM images of the hybrid structures supported on Ni foams prepared with urea plus thiourea at different Ni:Mn feeding mole ratios: (a) 0:1.05, (b) 0.3:0.75, (c) 0.4:0.65, (d) 0.5:0.55, (e) 0.6:0.45, (f) 0.8:0.25, (g) 0.9:0.15, (h) 1.05:0. The insets are the images of surface aggregates at higher magnification. (Heating process: 90°C 4 h + 110 ^{o}C 10 h).

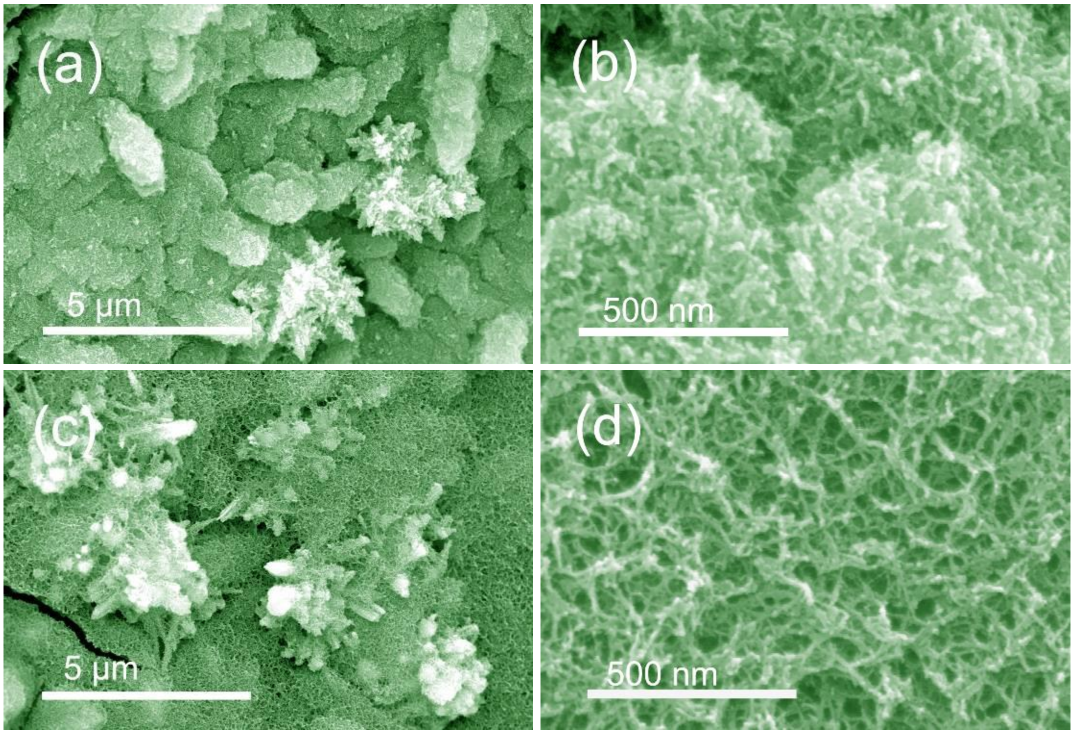

Figure S9. SEM images of the hybrid structures supported on Ni foam prepared with urea plus thiourea at 0.7:0.35 of Ni:Mn feeding mole ratio by using the heating process of (a, b) 90°C 14 h and (c, b) 110°C 14 h.

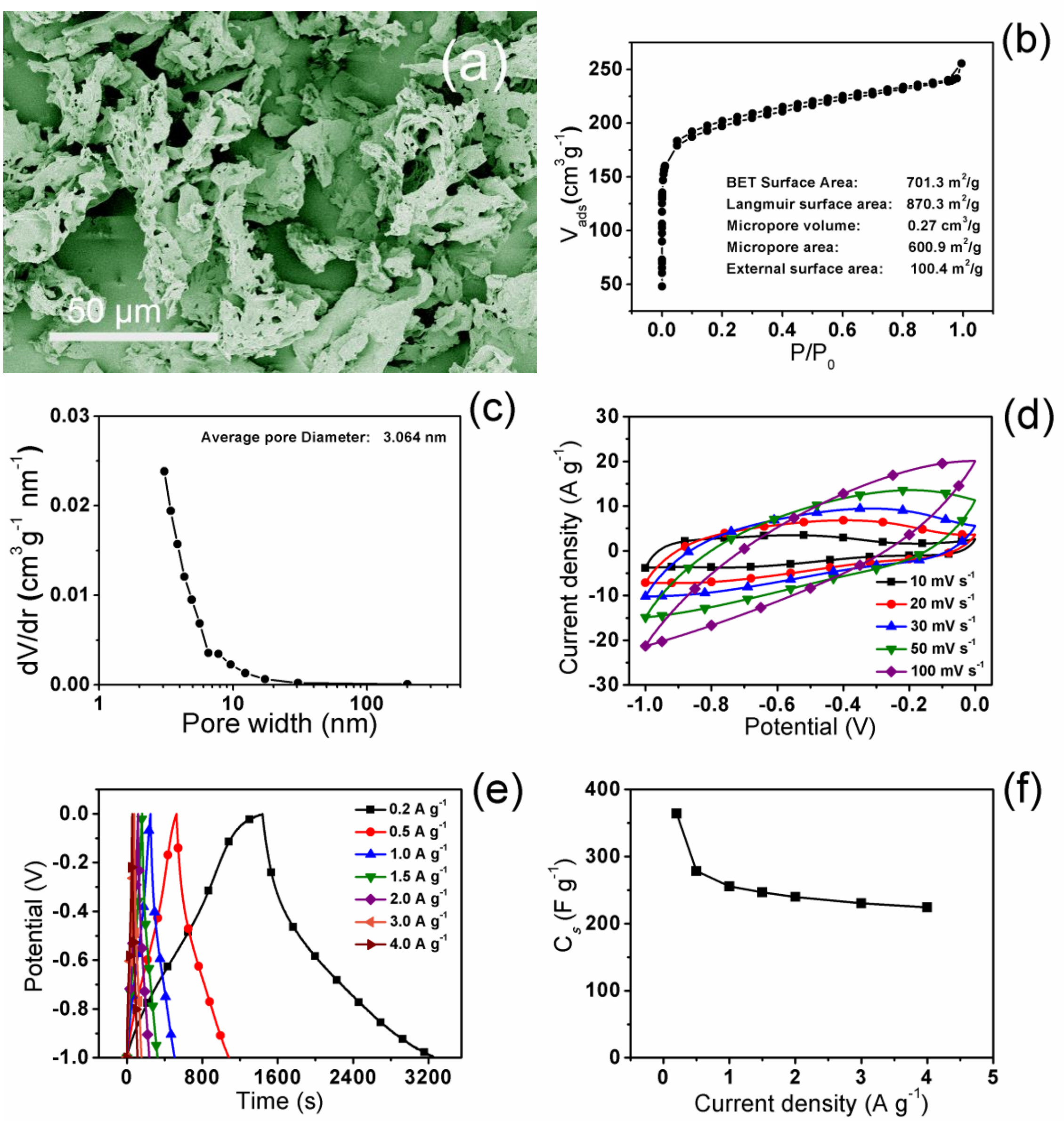

Figure S10. (a) A typical SEM image, (b) Nitrogen (77 K) adsorption/desorption isotherms, (c) BJH pore size distribution, (d) CV curves, (e) Galvanostatic charge-discharge curves and (f) C_s of as-synthesized AC.

Table S1. Comparison of structures, preparation methods, and maximum C_s of some reported NiMn-LDH and Ni_3S_2 based active materials and the present work.

Materials	Structures	Methods	Capacitance (F g^{-1})	Ref.
$NiCo_2S_4$ @NiMn-LDH/GS	Nanotube/ Nanosheet	Hydrothermal	205 (0.1 A g^{-1})	1
rGO paper based-NiMn-LDH/graphene	Nanosheet	Filtration process	217.8 (2 mA cm^{-2})	2
NiMn-LDH/rGO	Nanosheet	*In-situ* crystallization process	800 C g^{-1} (0.5 A g^{-1})	3
NiMn-LDH	Nanosheet	Reverse micelle method	881 (0.5 A g^{-1})	4
NiO/NiMn-LDH@NF	Nanosheet	Hydrothermal	937 (0.5 A g^{-1})	5
NiMn-LDH/rGO	Nanosheet	*In-situ* growth method	958 (1 A g^{-1})	6
NiMn-LDH/CNTs	Nanosheet	*In-situ* growth method	1044 (1 A g^{-1})	6
NiMn-LDH/CB	Nanosheet	*In-situ* growth method	1112 (1 A g^{-1})	6
NiMn-LDH/CNTs/rGO	Nanosheet	*In-situ* growth method	1268 (1 A g^{-1})	6
NiMn-LDH/carbon	Nanosheet	Hydrothermal + Annealing	1464 (0.5 A g^{-1})	7
NiMn-LDH@NF	Nanosheet	Wet-method	1511 (2.5 A g^{-1})	8
NiMn-LDH/rGO	Nanosheet	Co-precipitation	1635 (1 A g^{-1})	9
GOS/NiMn LDO	Nanosheet	Hydrothermal + Calcination	1648 (0.5 A g^{-1})	10
Carbon/NiMn-LDH@NF	Nanosheet	Hydrothermal	1916 (0.5 A g^{-1})	11
NiMn-LDH@CC	Nanosheet	Hydrothermal	2239 (5 mA cm^{-2})	12
NiMn-LDH/GO(S)	Nanosheet	Hydrothermal + Freeze drying	2246.63 (1 A g^{-1})	13
NiCoMn-LDH	Nanosheet	Hydrothermal	2420 (1 A g^{-1})	14
NiMn-LDH/CNT	Nanosheet	*In-situ* growth method	2960 (1.5 A g^{-1})	15

Ni_3S_2	Nanosheet-on-nanorods array	Hydrothermal	694 (3.45 A g^{-1})	16
Ni_3S_2	3D hierarchical dendrites	Hydrothermal	710.4 (2 A g^{-1})	17
Ni_3S_2	Nanoparticles	Mechanical alloying method/post heat treatment	911 (0.5 A g^{-1})	18
Ni_3S_2	Sheet-on-rod nanoarrays	Hydrothermal	1010 (2 mV s^{-1})	19
Ni_3S_2	Nanorod/nanowire arrays	Pre-oxidation/post hydrothermal	1051 (1.25 mA cm^{-2})	20
Ni_3S_2	Nanoflakes	Hydrothermal	1293 (5 mA cm^{-2})	21
Ni_3S_2	Nanosheet arrays	Hydrothermal	1370.4 (2 A g^{-1})	22
Ni_3S_2	Mushroom-like	Dissolution-precipitation route	1670 (1 A g^{-1})	23
Ni_3S_2	Thin Film	Hydrothermal	2230 (5 mA cm^{-2})	24
**NiMn-LDH@Ni_3S_2**	**Nanosheet@nanorod hybrid array**	**Hydrothermal**	**2703 (3 A g^{-1})**	**This work**

LDH: layered double hydroxide; NF: Ni foam; GOS: graphene oxide sponge; LDO: layered double oxide; rGO: reduced graphene oxide; CNTs: carbon nanotubes; CB: carbon black particle; CC: carbon cloth; GS: graphene sponge.

Table S2. Comparison of the maximum energy densities, corresponding average power densities and cycle stabilities of some reported NiMn-LDH and Ni_3S_2 based asymmetric supercapacitors, and the present work

Positive materials//negative materials	Energy density ($Wh\ kg^{-1}$)	Power density ($W\ kg^{-1}$)	Cycle stability	Ref.
NiMn-LDH/rGO//AC	–	–	72.2% (3 A g^{-1}, 1000 cycles)	6
NiMn-LDH/CNTs//AC	–	–	74.2% (3 A g^{-1}, 1000 cycles)	6
NiMn-LDH/CB//AC	–	–	70.7% (3 A g^{-1}, 1000 cycles)	6
GOS/NiMn-LDO// AC	–	–	86.7% (5 A g^{-1}, 6000 cycles)	10
$NiCo_2S_4$@NiMn-LDH/GS//VN/GS	–	–	84.5% (20 mA cm^{-2}, 5000 cycles)	1
NiMn-LDH@CC//EACC-3	–	–	110% (3 mA cm^{-2}, 10000 cycles)	12
NiMn-LDH/CNT//RGO/CNT	–	–	91% (10 A g^{-1}, 200 cycles)	15
NiMn-LDH/rGO//rGO	22.5	1000	–	3
NiO/NiMn-LDH//AC	27.8	401.3	–	5
NiMn-LDH/rGO//AC	33.8	850		9
Carbon-NiMn-LDH@NF //AC	37.7	378.13	84.2% (5 A g^{-1}, 5000 cycles)	11
NiMn-LDH/CNTs/rGO//AC	61.0	–	84.4% (3 A g^{-1}, 1000 cycles)	6
Ni_3S_2//carbon pen ink	8.2	214.6	93.1% (2.4 A g^{-1}, 3000 cycles)	25
Ni_3S_2//AC	34.6	150.4	85.7% (1 A g^{-1}, 1000 cycles)	22
Ni_3S_2//AC	36	400	86% (1 A g^{-1}, 1000 cycles)	18
Ni_3S_2//AC	41.8	155	76.6% (10 mA cm^{-2}, 2000 cycles)	26
Ni_3S_2//nitrogen-doped porous graphitic carbon	48.5	87.4	93.1% (40 mA cm^{-2}, 5000 cycles)	20
NiCoMn-LDH//rGO	57.4	749.9	90% (8 A g^{-1}, 2500 cycles)	14
**NiMn-LDH@Ni_3S_2//AC**	**57**	**738**	**79% (5 A g^{-1}, 4500 cycles)**	**This work**

LDH: layered double hydroxide; NF: Ni foam; AC: active carbon; GOS: graphene oxide sponge; LDO: layered double oxide; rGO: reduced graphene oxide; CNTs: carbon nanotubes; CB: carbon black particle; CC: carbon cloth; GS: graphene sponge; VN: vanadium nitride.

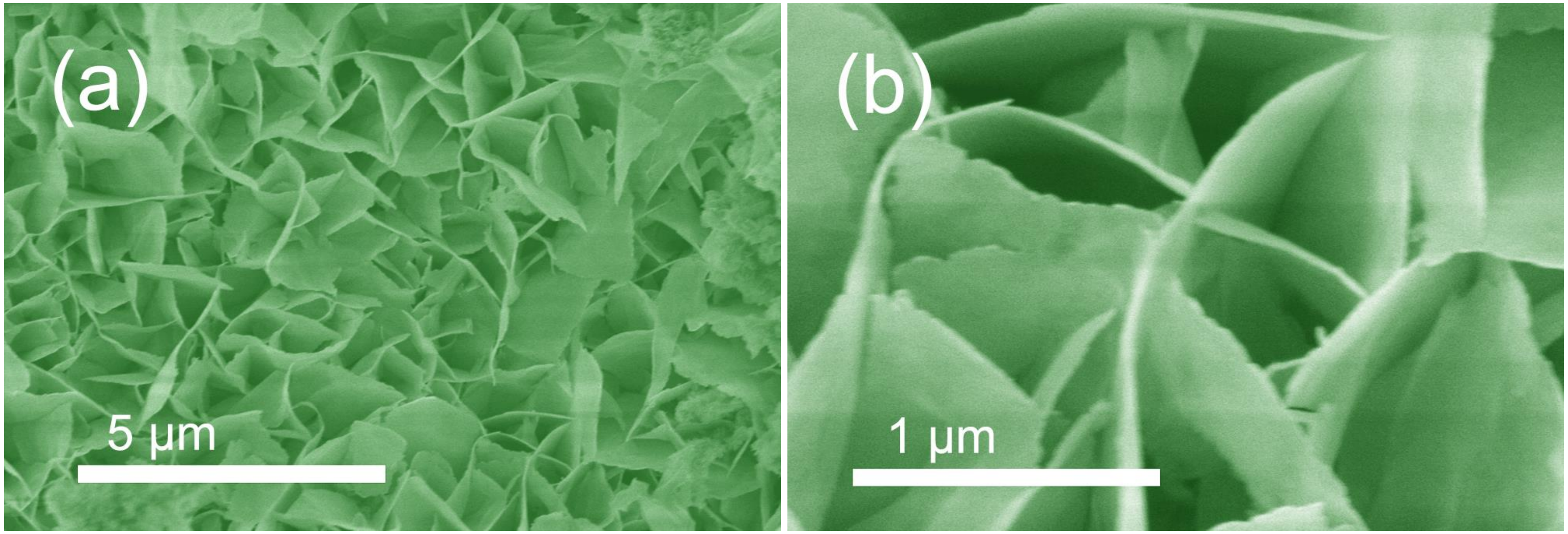

Figure S11. SEM images of the NiMn-LDH@Ni_3S_2 electrode after a long-term stability test

References for supporting information:

1. Wan, H. *et al.* Hierarchical Configuration of $NiCo_2S_4$ Nanotube@Ni–Mn Layered Double Hydroxide Arrays/Three-Dimensional Graphene Sponge as Electrode Materials for High-Capacitance Supercapacitors. *ACS Appl. Mater. Interfaces* **7**, 15840-15847 (2015).
2. Quan, W., Tang, Z. L., Wang, S. T., Hong, Y. & Zhang, Z. T. Facile preparation of free-standing rGO paper-based Ni-Mn LDH/graphene superlattice composites as a pseudocapacitive electrode. *Chem. Commun.* **52**, 3694-3696 (2016).
3. Padmini, M., Kiran, S. K., Lakshminarasimhan, N., Sathish, M. & Elumalai, P. High-performance Solid-state Hybrid Energy-storage Device Consisting of Reduced Graphene-Oxide Anchored with NiMn-Layered Double Hydroxide. *Electrochim. Acta* **236**, 359-370 (2017).
4. Sim, H. *et al.* Reverse Micelle Synthesis of Colloidal Nickel–Manganese Layered Double Hydroxide Nanosheets and Their Pseudocapacitive Properties. *Chem.-Eur. J.* **20**, 14880-14884 (2014).
5. Liu, P.-F. *et al.* A hierarchical NiO/NiMn-layered double hydroxide nanosheet array on Ni foam for high performance supercapacitors. *Dalton Trans.* **46**, 7388-7391 (2017).
6. Li, M., Liu, F., Zhang, X. B. & Cheng, J. P. A comparative study of Ni-Mn layered double hydroxide/carbon composites with different morphologies for supercapacitors. *Phys. Chem. Chem. Phys.* **18**, 30068-30078 (2016).
7. Lv, L. *et al.* Intercalation of Glucose in NiMn-Layered Double Hydroxide Nanosheets: an Effective Path Way towards Battery-type Electrodes with Enhanced Performance. *Electrochim. Acta* **216**, 35-43 (2016).
8. Guo, X. L. *et al.* Nickel-Manganese Layered Double Hydroxide Nanosheets Supported on Nickel Foam for High-performance Supercapacitor Electrode Materials. *Electrochim. Acta* **194**, 179-186 (2016).
9. Li, M., Cheng, J. P., Wang, J., Liu, F. & Zhang, X. B. The growth of nickel-manganese and cobalt-manganese layered double hydroxides on reduced graphene oxide for supercapacitor. *Electrochim. Acta* **206**, 108-115 (2016).
10. Chen, H. *et al.* Graphene-Karst Cave Flower-like Ni–Mn Layered Double Oxides Nanoarrays with Energy Storage Electrode. *Electrochim. Acta* **220**, 36-46 (2016).
11. Chen, H. *et al.* Carbon-coated Hierarchical Ni–Mn Layered Double Hydroxide Nanoarrays on Ni Foam for Flexible High-capacitance Supercapacitors. *Electrochim. Acta* **213**, 55-65 (2016).
12. Shi, L. *et al.* Flexible honeycomb-like NiMn layered double hydroxide/carbon cloth architecture for electrochemical energy storage. *Mater. Lett.* **175**, 275-278 (2016).
13. Chen, J., Wang, X., Wang, J. & Lee, P. S. Sulfidation of NiMn-Layered Double Hydroxides/Graphene Oxide Composites toward Supercapacitor Electrodes with Enhanced Performance. *Adv. Energy Mater.* **6**, 1501745 (2016).

14. Singh, S. *et al.* Tailoring the morphology followed by the electrochemical performance of NiMn-LDH nanosheet arrays through controlled Co-doping for high-energy and power asymmetric supercapacitors. *Dalton Trans.* **46**, 12876-12883 (2017).
15. Zhao, J. *et al.* Hierarchical NiMn Layered Double Hydroxide/Carbon Nanotubes Architecture with Superb Energy Density for Flexible Supercapacitors. *Adv. Funct. Mater.* **24**, 2938-2946 (2014).
16. Xiong, X. *et al.* One-step synthesis of architectural Ni_3S_2 nanosheet-on-nanorods array for use as high-performance electrodes for supercapacitors. *NPG Asia Mater.* **8**, e300 (2016).
17. Zhang, Z. *et al.* One-pot synthesis of hierarchically nanostructured Ni_3S_2 dendrites as active materials for supercapacitors. *Electrochim. Acta* **149**, 316-323 (2014).
18. Li, J.-J. *et al.* Mechanical alloying synthesis of Ni_3S_2 nanoparticles as electrode material for pseudocapacitor with excellent performances. *J. Alloy. Compd.* **656**, 138-145 (2016).
19. Chen, J. S., Gui, Y. & Blackwood, D. J. Self-supported phase-pure Ni_3S_2 sheet-on-rod nanoarrays with enhanced pseudocapacitive properties and high energy density. *J. Power Sources* **325**, 575-583 (2016).
20. Li, T. *et al.* Regulating the oxidation degree of nickel foam: a smart strategy to controllably synthesize active Ni_3S_2 nanorod/nanowire arrays for high-performance supercapacitors. *J. Mater. Chem. A* **4**, 8029-8040 (2016).
21. Krishnamoorthy, K., Veerasubramani, G. K., Radhakrishnan, S. & Kim, S. J. One pot hydrothermal growth of hierarchical nanostructured Ni_3S_2 on Ni foam for supercapacitor application. *Chem. Eng. J.* **251**, 116-122 (2014).
22. Huo, H., Zhao, Y. & Xu, C. 3D Ni_3S_2 nanosheet arrays supported on Ni foam for high-performance supercapacitor and non-enzymatic glucose detection. *J. Mater. Chem. A* **2**, 15111-15117 (2014).
23. Yang, B. *et al.* The growth and assembly of the multidimensional hierarchical Ni_3S_2 for aqueous asymmetric supercapacitors. *CrystEngComm* **17**, 4495-4501 (2015).
24. Ji, F. *et al.* Simple in-situ growth of layered Ni_3S_2 thin film electrode for the development of high-performance supercapacitors. *Appl. Surf. Sci.* **399**, 432-439 (2017).
25. Wen, J. *et al.* Flexible coaxial-type fiber solid-state asymmetrical supercapacitor based on Ni_3S_2 nanorod array and pen ink electrodes. *J. Power Sources* **324**, 325-333 (2016).
26. Li, J. *et al.* Controllable preparation of nanoporous Ni_3S_2 films by sulfuration of nickel foam as promising asymmetric supercapacitor electrodes. *Appl. Surf. Sci.* **420**, 919-926 (2017).
